# Supplementary material for: Implementation and adherence of routine pertussis vaccination (DTP) in a low-resource urban birth cohort
Source: BMJ Open. 2020 Dec 30;10(12):e041198. doi: 10.1136/bmjopen-2020-041198 (PMC7780521; doi:10.1136/bmjopen-2020-041198)
Supplement: Supplementary data [file bmjopen-2020-041198supp001.pdf]

Implementation and adherence of routine pertussis vaccination (DTP) in a low-resource urban birth cohort

Gunning et al.

November 13, 2020

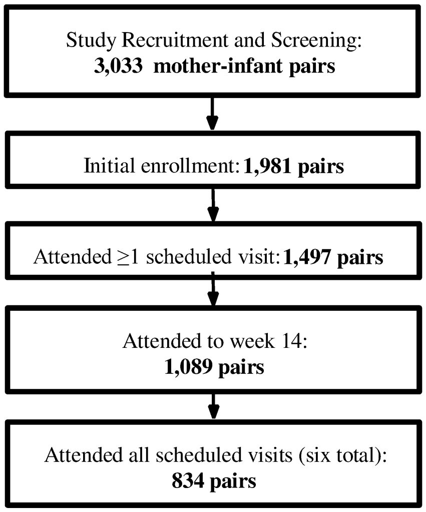

Figure S1: Study profile of birth cohort enrollment and attendance. Beyond eligibility and initial screening, the sole cause of cohort attrition was failure to attend one or more scheduled clinic visits.
