# Supplementary material for: Implementation and adherence of routine pertussis vaccination (DTP) in a low-resource urban birth cohort
Source: BMJ Open. 2020 Dec 30;10(12):e041198. doi: 10.1136/bmjopen-2020-041198 (PMC7780521; doi:10.1136/bmjopen-2020-041198)
Supplement: Supplementary data [file bmjopen-2020-041198supp002.pdf]

Implementation and adherence of routine pertussis vaccination (DTP) in a low-resource urban birth cohort

Gunning et al.

November 13, 2020

S1

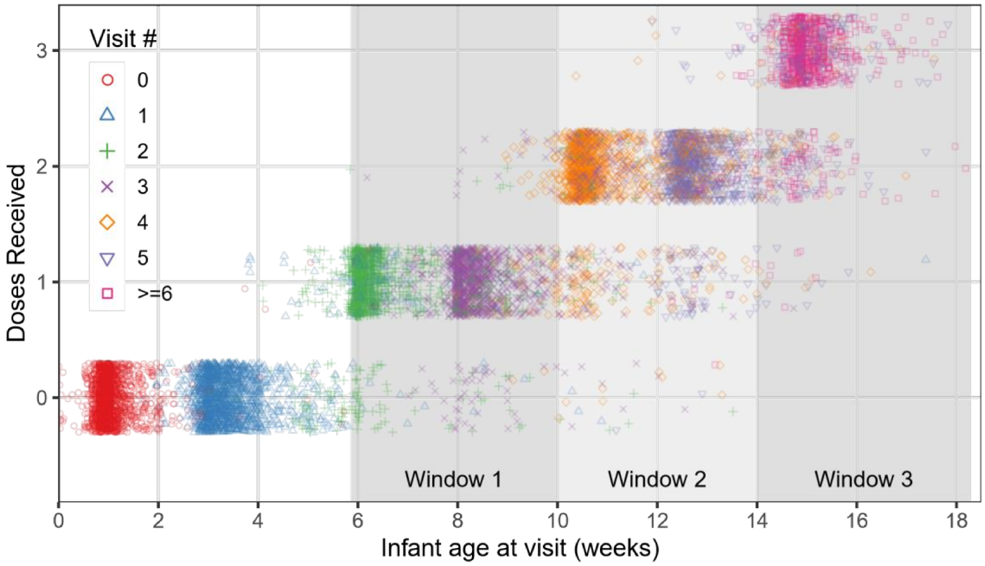

Figure S2: Infants were scheduled for 6 clinic visits over the course of the study. Figure shows the cumulative doses of DTP vaccine received per infant at each visit (color, shape) by infant age.
