# Supplementary material for: Implementation and adherence of routine pertussis vaccination (DTP) in a low-resource urban birth cohort
Source: BMJ Open. 2020 Dec 30;10(12):e041198. doi: 10.1136/bmjopen-2020-041198 (PMC7780521; doi:10.1136/bmjopen-2020-041198)
Supplement: Author's manuscript [file bmjopen-2020-041198.draft_revisions.pdf]

BMJ Open is committed to open peer review. As part of this commitment we make the peer review history of every article we publish publicly available.

When an article is published we post the peer reviewers' comments and the authors' responses online. We also post the versions of the paper that were used during peer review. These are the versions that the peer review comments apply to.

The versions of the paper that follow are the versions that were submitted during the peer review process. They are not the versions of record or the final published versions. They should not be cited or distributed as the published version of this manuscript.

BMJ Open is an open access journal and the full, final, typeset and author-corrected version of record of the manuscript is available on our site with no access controls, subscription charges or pay-per-view fees (http://bmjopen.bmj.com).

If you have any questions on BMJ Open's open peer review process please email info.bmjopen@bmj.com

BMJ Open

Implementation and adherence of routine pertussis vaccination (DTP) in a low-resource urban birth cohort

Journal:	*BMJ Open*
Manuscript ID	bmjopen-2020-041198
Article Type:	Original research
Date Submitted by the Author:	11-Jun-2020
Complete List of Authors:	Gunning, Christian ; University of Georgia, Odum School of Ecology<br>Mwananyanda, Lawrence; Right to Care<br>MacLeod, William; Boston University School of Public Health, Department of Global Health<br>Mwale, Magdalene; Right to Care<br>Thea, Donald; Boston University School of Public Health, Department of Global Health<br>Pieciak, Rachel; Boston University School of Public Health, Department of Global Health<br>Rohani, Pejman; University of Georgia, Odum School of Ecology<br>Gill, Christopher; Boston University School of Public Health, Department of Global Health
Keywords:	Public health < INFECTIOUS DISEASES, Community child health < PAEDIATRICS, Paediatric infectious disease & immunisation < PAEDIATRICS, Epidemiology < INFECTIOUS DISEASES

SCHOLARONE™
Manuscripts

I, the Submitting Author has the right to grant and does grant on behalf of all authors of the Work (as defined in the below author licence), an exclusive licence and/or a non-exclusive licence for contributions from authors who are: i) UK Crown employees; ii) where BMJ has agreed a CC-BY licence shall apply, and/or iii) in accordance with the terms applicable for US Federal Government officers or employees acting as part of their official duties; on a worldwide, perpetual, irrevocable, royalty-free basis to BMJ Publishing Group Ltd ("BMJ") its licensees and where the relevant Journal is co-owned by BMJ to the co-owners of the Journal, to publish the Work in this journal and any other BMJ products and to exploit all rights, as set out in our licence.

The Submitting Author accepts and understands that any supply made under these terms is made by BMJ to the Submitting Author unless you are acting as an employee on behalf of your employer or a postgraduate student of an affiliated institution which is paying any applicable article publishing charge ("APC") for Open Access articles. Where the Submitting Author wishes to make the Work available on an Open Access basis (and intends to pay the relevant APC), the terms of reuse of such Open Access shall be governed by a Creative Commons licence – details of these licences and which Creative Commons licence will apply to this Work are set out in our licence referred to above.

Other than as permitted in any relevant BMJ Author's Self Archiving Policies, I confirm this Work has not been accepted for publication elsewhere, is not being considered for publication elsewhere and does not duplicate material already published. I confirm all authors consent to publication of this Work and authorise the granting of this licence.

For peer review only

Implementation and adherence of routine pertussis vaccination (DTP) in a low-resource urban birth cohort

April 1, 2020

Authors: CE Gunning*, L Mwananyanda, WB Macleod, M Mwale, DM Thea, RC Pieciak, P Rohani, and CJ Gill

* Corresponding author (email: research@x14n.org)

For peer review only

Abstract

Introduction

Reliable information on rates of up-to-date coverage and timely administration of routine childhood immunizations is critical for guiding public health efforts worldwide, yet prospective observation of vaccination programs within individual communities is rare. Here we provide a longitudinal analysis of the directly-observed administration of a 3-dose primary vaccination series to infants in a low-resource community in Lusaka, Zambia.

Methods

Throughout 2015, we recruited a longitudinal birth cohort of mother/infant pairs (initial enrollment, 1,981 pairs; attending, 1,497 pairs) from the peri-urban informal settlement of Chawama compound, located in Lusaka, Zambia. We prospectively monitored the administration of scheduled Diphtheria-Tetanus-Pertussis (DTP) vaccinations across the first 14-18 weeks of life. We analyzed study attendance and vaccine coverage, both overall and stratified by age group. We employed Kaplan-Meier analyses to estimate delays in age-appropriate administration of vaccine doses. We also assessed schedule timing violations, including early and compressed dose administration.

Results

At study completion, first dose (DTP1) rates were high (89.9% of attending), whereas third dose completion (DTP3) rates were far lower (72.4%). Missed vaccinations and study dropout both contributed to the low DTP3 completion rates. DTP1 was administered very late (at or after 10 weeks) to 135 infants (9.0%). DPT1 was administered too early for 51 (3.4% of vaccinees) infants, and 66 (4.4%) received consecutive doses below the minimum recommended spacing of 28 days.

Conclusions

We observe substantial individual variation in the timing of early childhood DTP doses. Our results indicate that timely administration of both DTP1 and DPT3 remains a challenge in this community. These directly-observed, individual-based results provide an important counterpoint to more course-grained, survey-based national and province estimates of upto-

date vaccine coverage. This study also highlights the challenges of vaccine hesitancy and sub-optimal utilization of (no-cost) healthcare services in a low-resource urban setting.

Strengths and limitations of this study

- To our knowledge, this is the first prospective urban birth cohort study in a low-resource setting to directly document vaccinations at the place and time of administration.
- By following individuals across time, we are better able to assess healthcare utilization behavior relative to cross-sectional surveys.
- This study provides a detailed view of a single community across a year, and may not capture long-term trends or generalize to other populations.

Introduction

Estimates of vaccine coverage play a central role in epidemiology and public health, from identifying at-risk communities and age groups for supplementary immunization activities (SIAs) [1, 2] to their use in disease transmission models and subsequent inference [3–5]. Coverage estimates are also key indicators of national vaccination implementation success [6]. As such, high-quality vaccine coverage estimates are critical in shaping modern public health policy.

Estimates of childhood *up-to-date* vaccine coverage from cross-sectional studies (e.g., Demographic and Health Surveys (DHS) [7] and UNICEF [8]) have revealed numerous factors affecting vaccine administration in low- and middle-income countries (LMICs) [9–13]. However, these coarse-grained estimates can mask complexities in compliance and implementation. Particularly in LMICs, spatial and socioeconomic heterogeneities in coverage are common, as are delays in the timely administration of vaccine doses [9, 14]. Further, the reliability of surveys based on personal and informal records, i.e., vaccine cards and maternal recall, remains unclear [15].

With few exceptions [16, 17], detailed observations of childhood vaccinations during routine clinical care are lacking. Granular, community-based observations of individual patient histories can reveal temporal patterns in schedule adherence, including delays in age-appropriate vaccinations. Prospective observation of a cohort can also reveal if community-level DTP coverage differs substantially from regional coverage, and can help identify behavioral factors that affect the success of vaccine implementation programs, including vaccine hesitancy.

Here we provide a detailed analysis of infant vaccination in a sub-Saharan low-resource urban setting in Lusaka, Zambia. Throughout 2015, we followed a longitudinal birth cohort of mother-infant pairs from birth through 14 weeks of age. Our team prospectively documented administration of the primary 3-dose DTP series using an electronic data capture system [18]. We quantified the DTP series dose trajectory of individual infants, and assessed the proportion of infants with up-to-date vaccinations, or who missed one or more doses. We

For peer review only - http://bmjopen.bmj.com/site/about/guidelines.xhtml

evaluated compliance with published vaccine schedule guidelines, including minimum age restrictions and dose spacings.

In short, our use of directly-observed age-specific DTP administration data in a typical LMIC community clinic offers a unique window into vaccine implementation at the individual patient level that can help inform vaccine policy decisions globally.

Methods

Study design

The Southern Africa Mother Infant Pertussis Study (SAMIPS) was a longitudinal birth cohort study conducted in Lusaka, Zambia from March 2015-February 2016 to assess the incidence of pertussis in a representative population of otherwise healthy infants (details provided elsewhere [18]). Mother–infant pairs were enrolled from the population of the Chawama compound, a densely populated peri-urban slum near central Lusaka. The study goal was to capture all live births that occurred in Chawama compound during the study period. Infants were enrolled at the Chawama Primary Health Clinic (PHC) during their first scheduled postpartum well-child visit (at approximately 1 week old). As the only government-supported clinic in the community, Chawama PHC is the primary source of medical care for Chawama residents, allowing us maximize study reach. In addition, a public outreach campaign provided pregnant Chawama residents with information about the study prior to study initiation.

Enrollment eligibility required that infants were born after 37 weeks, weighed over 2500 grams, and were delivered without complications or apparent disease. Eligibility also required signed consent, Chawama residency (anticipated remaining in the community during study period), that the HIV status of mothers was known prior to delivery, and that HIV+ mothers were receiving prophylactic antiretroviral therapy at the time of delivery [18].

No financial compensation was provided to study participants. Regular clinic staff provided routine childhood vaccinations at no cost, while the SAMIPS study team provided all routine and acute medical care for participants for the study duration. Unscheduled clinic visits for acute healthcare needs are not considered here.

Enrolled infants were scheduled for six routine clinic visits at 2-3 week intervals through 14 weeks old (maximum, 18 weeks). At each clinic visit, nasopharyngeal (NP) swabs were obtained from both mother and infant, and possible pertussis symptoms were recorded. Administration of DTP doses 1-3 was scheduled at visits corresponding to 6, 10, and 14 weeks of age (see WHO Expanded Programme of Immunization (EPI) schedule [19]). DTP vaccinations were administered as a pentavalent combination (Pentavac, Serum Institute of India Limited, Pune, India) that included whole-cell pertussis, *Haemophilus influenzae* type B (HIB), and Hepatitis B. The pneumococcal conjugate vaccine (PCV10) was co-administered with DTP; oral rotavirus vaccine administration was scheduled for 6 and 10 weeks of age.

For peer review only

Patient and Public Involvement

This research was done without patient involvement. Patients were not invited to comment on the study design and were not consulted to develop patient relevant outcomes or interpret the results. Patients were not invited to contribute to the writing or editing of this document for readability or accuracy.

Assessment of Vaccine Administration

For protection against pertussis, the WHO recommends that infants receive a 3-dose primary series with a DTP-containing vaccine [19]. Guidelines specify a minimum administration age of 6 weeks for the first dose (DTP1), a minimum interval of 4 weeks between subsequent doses, and a target inter-dose interval of 4-8 weeks. In Zambia, pregnant mothers receive one or more doses of tetanus toxoid monovalent vaccine during pregnancy.

For this analysis, we use the Zambian 6-10-14 week EPI schedule for DTP to define target age windows in which infants should have received each DTP dose: window 1, 4169 days; window 2, 70-97 days; window 3, 98-126 days. Note that window 1 extends one day early to account for uncertainty the exact timing birth and vaccination. We tabulate the number of doses each infant received in each window, and at study completion. For example, an infant might receive no doses during window 1, and a dose in both windows 2 and 3, for a total of two doses at study completion.

Infants who received all scheduled doses to-date were categorized as *up-to-date*. However, once a window was missed, an infant was no longer up-to-date during subsequent age windows. Infants who received no doses by the end of the dose window were categorized as *unvaccinated*. Vaccinations that occurred >1 day prior to window 1 were classified as *early*. Vaccinations administered less than 4 weeks after a preceding dose were classified as *compressed*.

Data and Statistical Analyses

Administration of all DTP doses by clinic staff during the study period was directly observed and documented by study staff. All data were manually recorded on standardized data sheets, and digitized using a digital pen electronic data capture system (XCallibre, Durban, South Africa. https://www.xcallibre.com). Data were de-identified prior to analysis: each mother-infant pair was assigned a random date offset of +/-3 days, and all study dates were adjusted by this offset.

Mother-infant pairs were considered *attending* (on-study) from enrollment until their last recorded clinic visit during the study period. We exclude from further consideration individuals who enrolled in SAMIPS but did not attend any scheduled clinic visits.

We report the number of infants who received exactly (and at least) 0-3 doses by study completion, along with the percent of attending study infants in each of these groups.

We stratified dose outcomes by age window. For each window, we report the number of *unique* infants attending at least one scheduled visit. We also report the number of infants

For peer review only - http://bmjopen.bmj.com/site/about/guidelines.xhtml

who: received or missed a vaccine dose, received compressed or early vaccinations, and were up-to-date (along with respective percentages). Since attrition and irregular attendance are expected in cohort studies, we compute percentages relative to the number of unique infants in each window (above).

We perform a modified Kaplan-Meier analysis of vaccination delays [20, 14]. The standard Kaplan-Meier method assumes that non-attending individuals (i.e., right-censored) experience events at the same rate as attending individuals. Here we explore an alternative worst-case scenario where non-attending individuals receive no further vaccinations during the study period (subsequent to study drop-out). While subsequent make-up vaccinations may occur, our assumption of minimal off-study vaccination is plausible, as the Chawama clinic is the sole provider of DTP in this community, and study staff recorded all DTP doses there during the study period.

Results

Participation

Infants were enrolled in the study between March and December 2015 (1,981 out of 3,962 screened). Primary reasons for study exclusion include lack of consent (14.3%), low infant birth weight (7.9%), and community non-residence (5.9%). Enrollees that did not attend any subsequent clinic visits (484 total) were removed from this analysis, leaving 1,497 initially attending infants. We depict study participation over time of the remaining infants in Figure 1A. At the end of windows 1-3, 1,400 infants (window 1), 1,306 infants (window 2), and 1,086 infants (window 3) were still attending (see Figure S1 for study profile). The observed drop off in cohort size at the end of window 3 represents graduation from the study, as infants' final clinic visits (and DTP dose) were scheduled for approximately 14 weeks of age. Overall, the duration of study attendance was a median of 96 days (min=12d, max=120d).

Demographic Characteristics

Overall, the demographic characteristics of participants did not co-vary with duration of study attendance (see Table 1), including all enrolled individuals, individuals attending at least one scheduled visits (attending), and individuals attending all scheduled visits (completed). Mothers' median age was 25 years, regardless of attendance. Across enrolled mothers, 17.5% were HIV+, while slightly more attending mothers were HIV+ (19.3%). Infants' median birth weight was 3000 grams. Regardless of attendance, median household size was 1 infant of <1 year of age (including study infant) and 2 children of <5 years of age (including infants).

With few exceptions, demographic characteristics did not affect the relative risk of end-of-study outcomes: being unvaccinated, receiving at least one dose (DTP1), and receiving all three doses (DTP3) (Figure 2). Two exceptions were the lower relative risk of DTP3 (i.e., higher risk of incomplete vaccination) observed in infants of HIV+ mothers (RR=0.87, 95% CI: 0.76-0.99), and infants who received OPV at birth (RR=0.87, 95% CI: 0.77-0.96).

For peer review only

Table 1: **Demographic characteristics of participants** by duration of study attendance (columns), showing percentage or median, as appropriate. Interquartile range is shown in parentheses. **Attending** mother-infant pairs attended one or more scheduled clinic visit. **Completed** individuals attended all six scheduled visits.

	Duration of		
Parameter	Enrolled	Attending	Completed
Number Under Study	1981	1497	834
**Mothers**			
Married	90.2%	90.1%	90.3%
HIV+	17.5%	19.3%	18.6%
Median Age	25 (21, 29)	25 (22, 29)	25 (22, 30)
Median Infants In House (<1 year)	1 (1, 1)	1 (1, 1)	1 (1, 1)
Median Children In House (< 5 years)	2 (1, 2)	2 (1, 2)	2 (1, 2)
**Infants**			
Born at Chawama PHC	56.9%	56.7%	58.2%
Born at UTH	34.8%	35.5%	34.3%
Female Sex	46.9%	46.4%	46.4%
Received BCG at birth	46.4%	46.6%	46.2%
Received OPV at birth	33.4%	32.4%	27.7%
Median Birth Weight (kg)	3 (2.8, 3.3)	3 (2.8, 3.3)	3.1 (2.8, 3.3)

Scheduled Doses

The administration of DTP doses to individual participants is shown in Figure 1B (for a detailed view of all participant clinic visits, see Figure S2). We also aggregate these data by week, highlighting that most infants received each DTP dose within a week of the target schedule of 6, 10, and 14 weeks of age (Figure 1C). However, deviations from the planned schedule were common. For example, early DTP vaccinations were administered to 51 infants prior to window 1 (Figure 4A), before which no vaccinations should have occurred (save for doses of the oral polio and BCG vaccines, administered to some infants at birth; see Table 1). Similarly, the spread of events to the right of 6, 10 and 14 weeks represents administration of vaccines after the scheduled delivery date. Two general trends are apparent across the three scheduled doses. With each subsequent dose: 1), the average time of administration drifts farther to the right of (later than) the target schedule; and 2) the clustering of administration times becomes more dispersed. Both trends reinforce the general concept that events occurring earlier in life have inevitable down-stream effects on subsequent vaccinations.

Infants who missed a dose and later received a subsequent dose on (or near) schedule are also evident in Figures 1B-C. For example, 98 infants received their first DTP dose at age 10 weeks, while 160 infants received a second DTP dose at age 14-15 weeks (Figure 1C). Since the study did not follow participants beyond age 16 weeks (with few exceptions), it remains unclear whether (and when) catch-up of any doses missed during the study might have occurred.

For peer review only - http://bmjopen.bmj.com/site/about/guidelines.xhtml

Vaccination outcomes at the completion of the study (total doses received) are summarized in Table 2. The number of doses received by infants in each age windows is further detailed in Table 3. Overall, approximately 8.2% of 1,497 attending infants had not received any DTP vaccinations at the conclusion of the study. A similar pattern was observed within each window, where infants who visited the clinic in windows 1-3 nonetheless missed scheduled vaccinations at a rate of 12.2% (window 1), 14.0% (window 2), and 10.4% window 3 (Table 4).

Across the study, the up-to-date rate fell from 89.9% of attending infants during window 1 to 72.2% during window 3 (Table 4). The unvaccinated rate also fell from 18.5% during window 1 to 11.4% during window 3. At study conclusion, and adjusting for the population of infants who remained in the cohort up to the beginning of window 3 (and thus had the opportunity to receive each of the three scheduled doses, see Figure 1A), 11.4% of infants (123/1,076) had not received any doses of DTP.

Table 2: **Vaccine status at study completion.** Number of infants that received at least (or exactly) 0-3 doses of DTP at study completion. Percentages are rounded (out of 1497 attending infants).

N	Exactly N Doses (%)	At Least N Doses (%)
0	123 (8.2)	1497 (100.0)
1	190 (12.7)	1374 (91.8)
2	393 (26.3)	1184 (79.1)
3	791 (52.8)	791 (52.8)

Table 3: **Doses received**, showing number of unique infants that received DTP1-3 by the *end* of each age window (columns, in days). Bottom row shows total doses administered in each window (some infants received more than one dose per window).

	Early	Window 1	Window 2	Window 3
Received	[0,41)	[41,70)	[70,98)	[98,128)
DTP1	51	1188	118	17
DTP2		24	990	170
DTP3			12	779
Any	51	1212	1120	966

For peer review only - http://bmjopen.bmj.com/site/about/guidelines.xhtml

Table 4: **Vaccinations stratified by age window,** showing number of unique infants *within* each age window. **Visits:** infants attending ≥1 scheduled visit. **Received, Missed:** infants that received (or did not receive) a DTP dose. **Compressed:** infants vaccinated <4 weeks after previous dose (see Fig. 4B). **Up-to-date:** infants that received the targeted N^{th} dose in this window. **Unvaccinated:** infants that received zero total doses by end of window. Percentages are rounded (out of **Visited**).

Age Window	Target (N)	Visits	Received (%)	Missed (%)	Compressed (%)	Up-to-date (%)	Unvaccinated (%)
**[41,70)**	**1**	1370	1203 (87.8%)	167 (12.2)	-	1231 (89.9)	253 (18.5)
**[70,98)**	**2**	1295	1114 (86.0%)	181 (14.0)	40 (3.1)	1011 (78.1)	139 (10.7)
**[98,128)**	**3**	1077	964 (89.5%)	113 (10.5)	26 (2.4)	779 (72.3)	123 (11.4)

Vaccine timing

A timely initial dose plays an important role in protecting infants from preventable morbidity and mortality [21]; prior to DTP1, study infants' protection against pertussis depended on unknown but presumably low and waning maternally-derived immunity. We observed 135 attending infants (9.0%) that received DTP1 at 10 weeks of age or greater (Table 3 and Figure 3), while 17 (1.1%) received DTP1 at 14 weeks of age or greater.

The Kaplan-Meier analysis revealed a modest median delay in the timely administration of DTP1 (1 day), though the median delay grew across primary series to 5 days (DTP2) and 13 days (DTP3). However, many infants experienced more substantial delays: for DTP1 the upper decile delay (slowest 10%) was 38 days, and for DTP2 the upper quantile delay (slowest 25%) was 36 days, while 47.2% of attending infants did not receive DTP3 during the study.

By comparison, schedule violations involving early and compressed doses were minor. DTP1 was administered too early to 51 infants (3.4% of attending), with most of these doses administered 2-7 days early (at age 35-40 days) (Figure 4A). The median spacing between subsequent doses (31 days) slightly exceeded the minimum recommended spacing of 28 days (Figure 4B). A small minority of infants experienced dose compression between doses 1-2 and/or doses 2-3: 66 infants received doses spaced less than 25 days apart, while 38 infants received doses less than 20 days apart.

Discussion

This study offers a descriptive analysis of prospectively-collected, carefully documented observations of DTP vaccinations administered in an urban African public health clinic. Overall, we found that most infants received the full DTP3 series in a timely manner, indicating successful initiation into the EPI. This is remarkable given that Chawama's EPI implementation lacks direct client outreach or other supportive interventions. However, we were concerned by higher than anticipated rate of missed (and delayed) doses: at study completion, only 52.5% of (initially) attending infants had received all three doses, while 8.2% of attending infants lacked records for any DTP vaccinations. In short, our findings suggest that access to (no-cost) healthcare is not the sole limiting factor in DTP uptake in Chawama compound.

For peer review only - http://bmjopen.bmj.com/site/about/guidelines.xhtml

While not directly comparable with our results, WHO-UNICEF estimated that Zambia's 2015 up-to-date DTP3 coverage was 90% [22], much higher than our estimates. However, our DTP1 rates at study completion (91.8%) were proportionally closer to corresponding WHO-UNICEF estimates (97%) and 2013-2014 Zambia DHS estimates for Lusaka province (97.8%) [23] (though the ceiling effect leaves little range for observable differences here). Overall, our results suggest that spatial heterogeneity and delays in timely vaccination both warrant further attention in Zambia.

The longitudinal nature of this study allowed us to resolve key implementation features that cross-sectional or survey-based approaches cannot resolve, including dose timing. We also gained insights into how individuals' behavior affected population-level coverage rates. For example, the small change in unvaccinated infants attending window 2 (139, 10.7%) and window 3 (123, 11.4%) suggests that doses are not skipped randomly by study participants. Rather, a core group of individuals appears to have regularly attend clinic visits while opting out of (no-cost) routine vaccines, despite encouragement by our study team. Indeed, earlier qualitative work in this cohort suggests that vaccine hesitancy plays a non-trivial role here [24].

Following a birth cohort across almost five months of life proved challenging in this setting. We experienced high rates of initial non-participation amongst eligible infants (484 out of 1,981 initial enrollees), as well as ongoing attrition of the cohort throughout the study. Indeed, only 71.9% of attending infants (1,077, Figure 1) participated in the study up to an age of 14 weeks (98 days), and were thus available to receive all scheduled DTP doses during the study. On the other hand, study involvement could have introduced an upwards bias on clinic visit behavior and DTP uptake relative to this population's background, non-study health care behavior.

Two unresolved questions remain: 1) whether infants who left the cohort early were subsequently vaccinated or were vaccinated elsewhere off study, and 2) whether attending infants who missed scheduled doses later received catch-up doses, e.g., at subsequent well-child visits. In Lusaka, catch-up of missed doses is available at monthly clinic visits and two targeted "child health weeks" (June and November). However, these supplemental vaccination campaigns are implemented without respect to known vaccination compliance (as documented in each child's under five card), and thus do not systematically target children who missed doses during the primary series. Moreover, after the final study visit (at age 14-16 weeks), the next routine well child visit is not until nine months of age, when the measles vaccine dose is given. As such, even when supplemental vaccination occurs, the subsequent delays in timely vaccination may be substantial.

While beyond the scope of our study, a cross-sectional seroconversion survey of young children (e.g., 1-5 years of age) could help quantify the frequency of late or absent vaccination. For example, seropositivity for tetanus antibodies could provide a robust indicator of (at least) DTP1 uptake, as tetanus toxoid is highly immunogenic and elicits durable immunity [25].

For peer review only - http://bmjopen.bmj.com/site/about/guidelines.xhtml

Despite lingering uncertainties, our analyses raise broad questions about pertussis control in low-resource settings, including herd immunity and epidemic preparedness. Crowded living situations can facilitate pertussis transmission relative to less dense regions [26, 27], while the scarcity of macrolide treatments in this community may yield longer-lasting infections [28]; both effects could raise the critical vaccination threshold required for herd immunity [29]. Further, lower DTP coverage here relative to Lusaka province is consistent with the previously-described impacts of socioeconomic status [10–13]. Together, these forces could allow dense informal settlements to serve as foci for more widespread pertussis epidemics. Our results also highlight the potential public health consequences of such an epidemic. We observed 253 infants (16.9% of attending) who remained unvaccinated at age 10 weeks, and were at elevated risk of severe disease, hospitalization, and death [30, 31]. Yet low-resource settings are ill-prepared to treat severe cases or high case loads. Indeed, Chawama Compound has 10 hospital beds to serve a population of more than 150,000 persons.

These results have additional implications for the full package of vaccines administered via the EPI schedule, as failure to receive a scheduled DTP dose may also indicate failure to receive additionally scheduled vaccinations. For example, both the pneumococcal conjugate and rotavirus vaccines are commonly co-administered with DTP. We expect that marginal improvements in EPI implementation would be highly impactful to public health outcomes, particularly in under-served communities, and that detailed descriptions of when and why EPI schedule violations occur will help identify and remediate existing barriers to vaccine uptake in these communities.

In summary, this study provides a uniquely granular view of routine DTP implementation in a low-resource urban African setting that highlights individual behavior. Our data suggest that delayed age of first vaccination, along with possible vaccine hesitancy and loss of herd immunity, poses a significant public health risk to this community, echoing calls to strengthen EPI programs in low-resource urban settings [32, 33].

Competing Interests

The authors declare no competing interests.

Data Sharing

De-identified data will be made available in a public, open access repository (Data Dryad) prior to publication. The R code used to produce all analyses and figures is available on request.

Author Contributions

Conceived study: CJG. Designed study: CJG, CEG. Conducted analysis, created figures and tables: CEG. Contributed to and interpreted analysis: CEG, WM, PR, CJG. Wrote manuscript: CEG, PR, CJG. Contributed to manuscript revisions: All authors.

Funding

The Bill & Melinda Gates Foundation funded the initial SAMIPS cohort (OPP 1105094). The National Institutes of Health/National Institute of Allergy and Infectious Diseases supported all subsequent analyses, including this analysis, using the SAMIPS database (1R01 AI133080). The study sponsors had no role in study design, analysis, or publication.

For peer review only - http://bmjopen.bmj.com/site/about/guidelines.xhtml

References

[1] Marita Zimmermann, Kurt Frey, Brittany Hagedorn, AJ Oteri, Abdulazeez Yahya, Maimuna Hamisu, Fred Mogekwu, Faisal Shuaib, Kevin A McCarthy, and Guillaume Chabot-Couture. Optimization of frequency and targeting of measles supplemental immunization activities in Nigeria: A cost-effectiveness analysis. *Vaccine*, 37 (41):6039–6047, 2019.

[2] Maria Joyce U Ducusin, Maricel de Quiroz-Castro, Sigrun Roesel, Luzviminda C Garcia, Dulce Cecilio-Elfa, W William Schluter, James L Goodson, and Eugene Lam. Using the World Health Organization measles programmatic risk assessment tool for monitoring of supplemental immunization activities in the Philippines. *Risk Analysis*, 37(6):1082–1095, 2017.

[3] Manoj Gambhir, Thomas A Clark, Simon Cauchemez, Sara Y Tartof, David L Swerdlow, and Neil M Ferguson. A change in vaccine efficacy and duration of protection explains recent rises in pertussis incidence in the United States. *PLoS Comput Biol*, 11(4):e1004138, 2015.

[4] Yoon Hong Choi, Helen Campbell, Gayatri Amirthalingam, Albert Jan Van Hoek, and Elizabeth Miller. Investigating the pertussis resurgence in England and Wales, and options for future control. *BMC medicine*, 14(121): 1–11, 2016.

[5] Matthieu Domenech de Cell`es, Felicia M. G. Magpantay, Aaron A. King, and Pejman Rohani. The impact of past vaccination coverage and immunity on pertussis resurgence. *Science Translational Medicine*, 10(434), 2019. ISSN 1946-6234. doi: 10.1126/scitranslmed.aaj1748. URL https://stm.sciencemag.org/content/10/434/eaaj1748.

[6] Jean-Marie Okwo-Bele and Thomas Cherian. The Expanded Programme on Immunization: a lasting legacy of smallpox eradication. *Vaccine*, 29:D74–D79, 2011.

[7] Madeleine Short Fabic, YoonJoung Choi, and Sandra Bird. A systematic review of demographic and health surveys: data availability and utilization for research. *Bulletin of the World Health Organization*, 90:604–612, 2012.

[8] Anthony Burton, Roeland Monasch, Barbara Lautenbach, Marta Gacic-Dobo, Maryanne Neill, Rouslan Karimov, Lara Wolfson, Gareth Jones, and Maureen Birmingham. WHO and UNICEF estimates of national infant immunization coverage: methods and processes. *Bulletin of the World Health Organization*, 87:535–541, 2009. URL https://www.who.int/bulletin/volumes/87/7/08-053819/en/.

[9] Andrew Clark and Colin Sanderson. Timing of children's vaccinations in 45 low-income and middle-income countries: an analysis of survey data. *The Lancet*, 373(9674):1543–1549, 2009.

[10] Mary-Christine Sullivan, Ayalew Tegegn, Fasil Tessema, Sandro Galea, and Craig Hadley. Minding the immunization gap: family characteristics associated with completion rates in rural Ethiopia. *Journal of Community Health*, 35(1):53–59, 2010.

[11] Ahmad Reza Hosseinpoor, Nicole Bergen, Anne Schlotheuber, Marta Gacic-Dobo, Peter M Hansen, Kamel Senouci, Ties Boerma, and Aluisio JD Barros. State of inequality in Diphtheria-Tetanus-Pertussis immunisation coverage in low-income and middle-income countries: a multi-country study of household health surveys. *The Lancet Global Health*, 4(9): e617–e626, 2016.

[12] Jin-Won Noh, Young-mi Kim, Nabeel Akram, Ki-Bong Yoo, Jumin Park, Jooyoung Cheon, Young Dae Kwon, and Jelle Stekelenburg. Factors affecting complete and timely childhood immunization coverage in Sindh, Pakistan; a secondary analysis of cross-sectional survey data. *PloS one*, 13(10):e0206766, 2018.

[13] Ikuma Nozaki, Masahiko Hachiya, and Tomomi Kitamura. Factors influencing basic vaccination coverage in Myanmar: secondary analysis of 2015 Myanmar demographic and health survey data. *BMC public health*, 19(1): 242, 2019.

[14] Manas K Akmatov and Rafael T Mikolajczyk. Timeliness of childhood vaccinations in 31 low and middle-income countries. *J Epidemiol Community Health*, 66(7):e14–e14, 2012.

[15] Melody Miles, Tove K Ryman, Vance Dietz, Elizabeth Zell, and Elizabeth T Luman. Validity of vaccination cards and parental recall to estimate vaccination coverage: a systematic review of the literature. *Vaccine*, 31 (12):1560–1568, 2013.

[16] Shankar Prinja, Madhu Gupta, Amarjeet Singh, and Rajesh Kumar. Effectiveness of planning and management interventions for improving age-appropriate immunization in rural India. *Bulletin of the World Health Organization*, 88:97–103, 2010. doi: 10.2471/BLT.08.059543.

For peer review only - http://bmjopen.bmj.com/site/about/guidelines.xhtml

[17] Ayebo Evawere Sadoh, Damian Uchechukwu Nwaneri, Bamidele Charity Ogboghodo, and Wilson Ehidiamen Sadoh. Effect of introduction of pentavalent vaccine as replacement for Diphtheria–Tetanus–Pertussis and Hepatitis B vaccines on vaccination uptake in a health facility in Nigeria. *Vaccine*, 34(24):2722–2728, 2016.

[18] Christopher J Gill, Lawrence Mwananyanda, William MacLeod, Geoffrey Kwenda, Magdalene Mwale, Anna L Williams, Kazungu Siazeele, Zhaoyan Yang, James Mwansa, and Donald M Thea. Incidence of severe and non-severe pertussis among HIV-exposed and-unexposed Zambian infants through 14 weeks of age: results from the southern Africa mother infant pertussis study (SAMIPS), a longitudinal birth cohort study. *Clinical Infectious Diseases*, 63(suppl 4):S154–S164, 2016.

[19] World Health Organization. Pertussis vaccines: WHO position paper—august 2015. *Weekly Epidemiological Record*, 90(35):433–460, 2015.

[20] B Laubereau, M Hermann, HJ Schmitt, J Weil, and R von Kries. Detection of delayed vaccinations: a new approach to visualize vaccine uptake. *Epidemiology & Infection*, 128(2):185–192, 2002.

[21] Zhiyun Chen and Qiushui He. Immune persistence after pertussis vaccination. *Human vaccines & immunotherapeutics*, 13(4):744–756, 2017.

[22] WHO. WHO-UNICEF estimates time series for Zambia (ZMB). http://apps.who.int/immunization_monitoring/globalsummary/estimates?c=ZMB. Accessed: 2019-10-01.

[23] Central Statistical Office/Zambia, Ministry of Health/Zambia, University of Zambia Teaching Hospital Virology Laboratory, University of Zambia Department of Population Studies, Tropical Diseases Research Centre/Zambia, and ICF International. Zambia demographic and health survey 2013-14. http://dhsprogram.com/ pubs/pdf/FR304/FR304.pdf, 2015.

[24] Anna Larson Williams, Rachel Mitrovich, Lawrence Mwananyanda, and Chris Gill. Maternal vaccine knowledge in low-and middle-income countries—and why it matters. *Human vaccines & immunotherapeutics*, 15(2):283– 286, 2019.

[25] Yanping Li, Rong Cheng Li, Qiang Ye, Changgui Li, You Ping Liu, Xiao Ma, Yanan Li, Hong Zhao, Xiaoling Chen, Deepak Assudani, et al. Safety, immunogenicity and persistence of immune response to the combined Diphtheria, Tetanus, acellular Pertussis, poliovirus and Haemophilus influenzae type b conjugate vaccine (dtpaipv/hib) administered in Chinese infants. *Human vaccines & immunotherapeutics*, 13(3):588–598, 2017.

[26] Pamela Rendi-Wagner, Joshua Tobias, Larisa Moerman, Sophy Goren, Ravit Bassal, Manfred Green, and Dani Cohen. The seroepidemiology of Bordetella pertussis in Israel—estimate of incidence of infection. *Vaccine*, 28 (19):3285–3290, 2010.

[27] FMG Magpantay and P Rohani. Dynamics of pertussis transmission in the United States. *American journal of epidemiology*, 181(12):921–931, 2015.

[28] Centers for Disease Control and Prevention. Recommended antimicrobial agents for the treatment and postexposure prophylaxis of pertussis: 2005 CDC guidelines. *MMWR*, 54(RR-14):1–16, 2005.

[29] R.M. Anderson and R.M. May. *Infectious diseases of humans*. Oxford University Press Oxford, 1991.

[30] Veronica Kolos, Robert Menzies, and Peter McIntyre. Higher pertussis hospitalization rates in indigenous Australian infants, and delayed vaccination. *Vaccine*, 25(4):588–590, 2007.

[31] Helen Campbell, Gayatri Amirthalingam, Nick Andrews, Norman K Fry, Robert C George, Timothy G Harrison, and Elizabeth Miller. Accelerating control of pertussis in England and Wales. *Emerging infectious diseases*, 18 (1):38, 2012.

[32] Md Jasim Uddin, Charles P Larson, Elizabeth Oliveras, AI Khan, MA Quaiyum, and Nirod Chandra Saha. Child immunization coverage in urban slums of Bangladesh: impact of an intervention package. *Health policy and planning*, 25(1):50–60, 2009.

[33] Martin K Mutua, Elizabeth Kimani-Murage, and Remare R Ettarh. Childhood vaccination in informal urban settlements in Nairobi, Kenya: who gets vaccinated? *BMC public health*, 11(1):6, 2011.

For peer review only

Figure Captions

Figure 1. **A:** Study participation by infant age. Shaded regions show scheduled administration of DTP doses 1-3. **B:** Timeline of DTP dose administration (see also Figure S2). C: Number of vaccinations per week, by dose number.

Figure 2. Relative risk of vaccination outcome (columns) by demographic covariate (y-axis), showing 95% CI. Select p-values are shown above estimates.

Figure 3. Kaplan-Meier analysis: estimated dose administration probability for DTP doses 1-3, assuming no vaccination in infants lost to follow-up. Vertical grey lines show dose target age; intersections with horizontal dashed lines show the median, upper quantile (slowest 25%) and upper decile (slowest 10%) delays for each dose. Tables show number of infants at select ages.

Figure 4. **A:** Number of early vaccinations (DTP1) by infant age. **B:** Number of vaccinations by dose spacing (time since previous dose), split by dose number (columns). Rows show compressed versus appropriate spacing (rows).

For peer review only

For peer review only - http://bmjopen.bmj.com/site/about/guidelines.xhtml

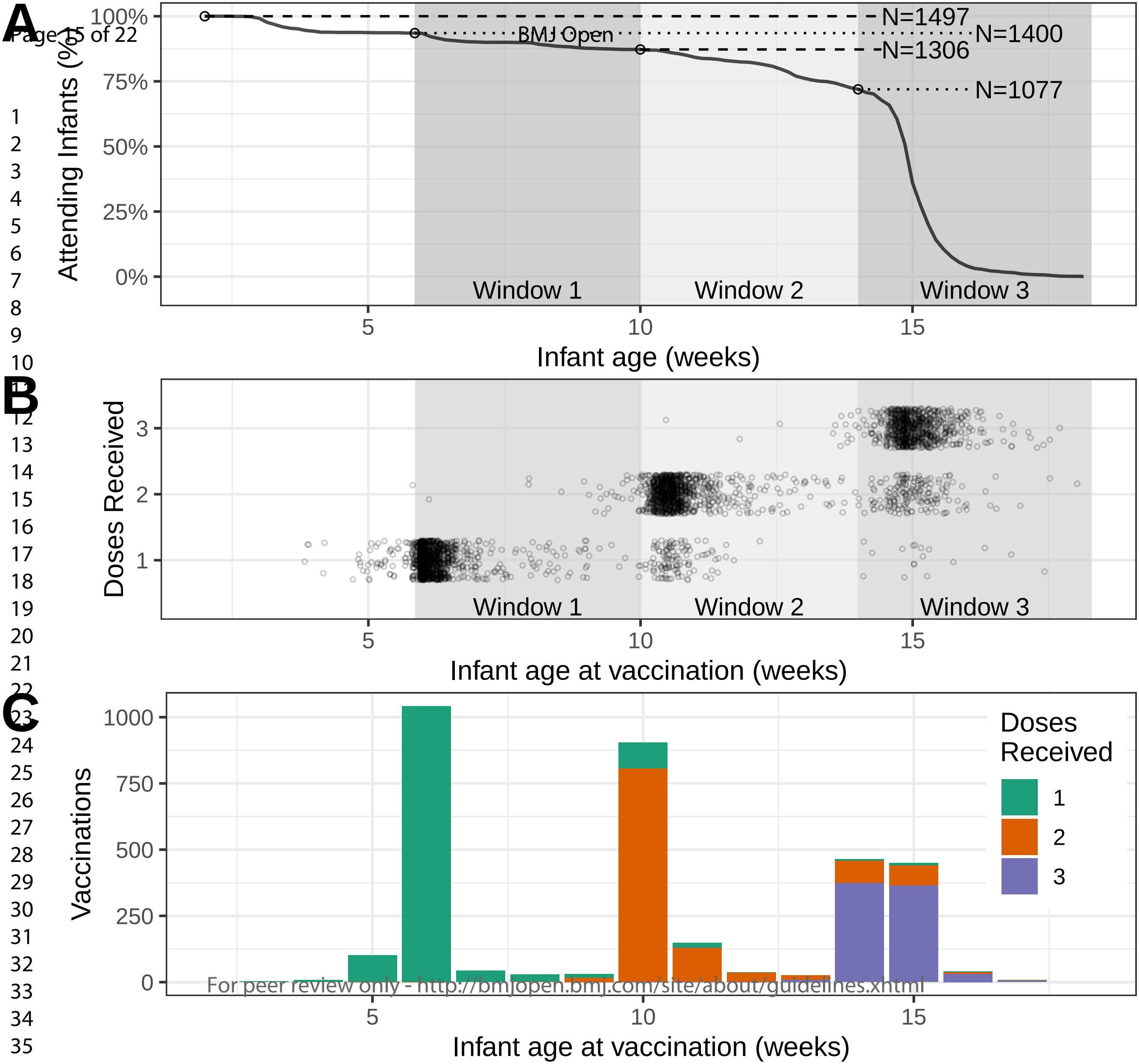

For peer review only - http://bmjopen.bmj.com/site/about/guidelines.xhtml

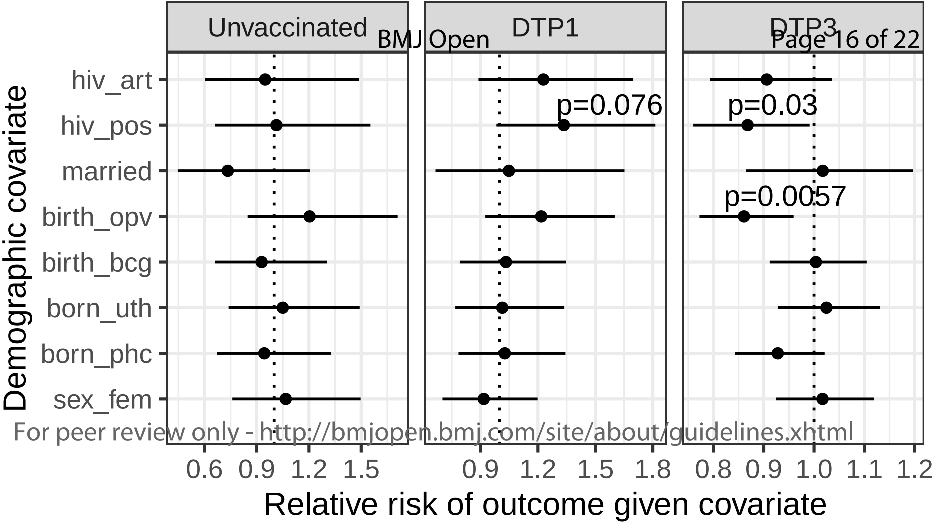

For peer review only - http://bmjopen.bmj.com/site/about/guidelines.xhtml

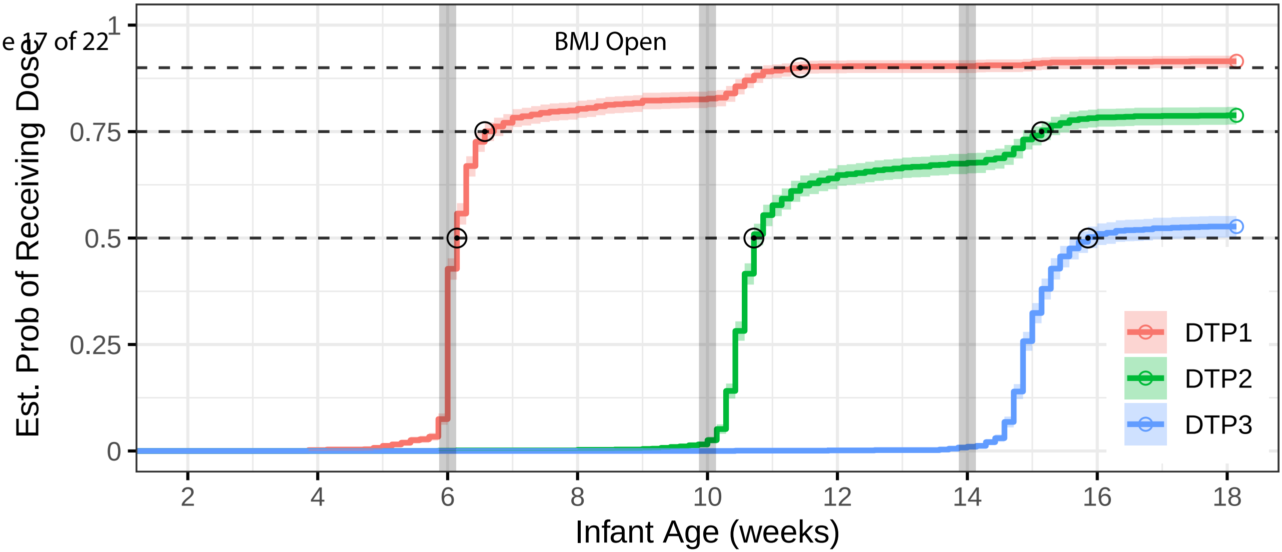

Attending, Awaiting Dose:

	2	4	6	8	10	12	14	16	18
DTP1	1496	1493	1384	300	261	145	144	130	127
DTP2	1497	1497	1496	1495	1474	539	487	327	318
DTP3	1497	1497	1497	1497	1497	1495	1485	745	708

Received Dose:

	2	4	6	8	10	12	14	16	18
DTP1	0	3	640	1202	1238	1351	1352	1366	1369
DTP2	0	0	1	4	38	970	1013	1173	1180
DTP3	0	0	0	0	0	2	15	763	789

Lost to Follow−up:

	2	4	6	8	10	12	14	16	18
DTP1	0	93	96	100	104	117	124	127	127
DTP2	0	92	117	163	197	234	273	310	316
DTP3	0	[illegible]	[illegible]	[illegible]	[illegible]	[illegible]	[illegible]	688	707

Infant Age (weeks)

For peer review only - http://bmjopen.bmj.com/site/about/guidelines.xhtml

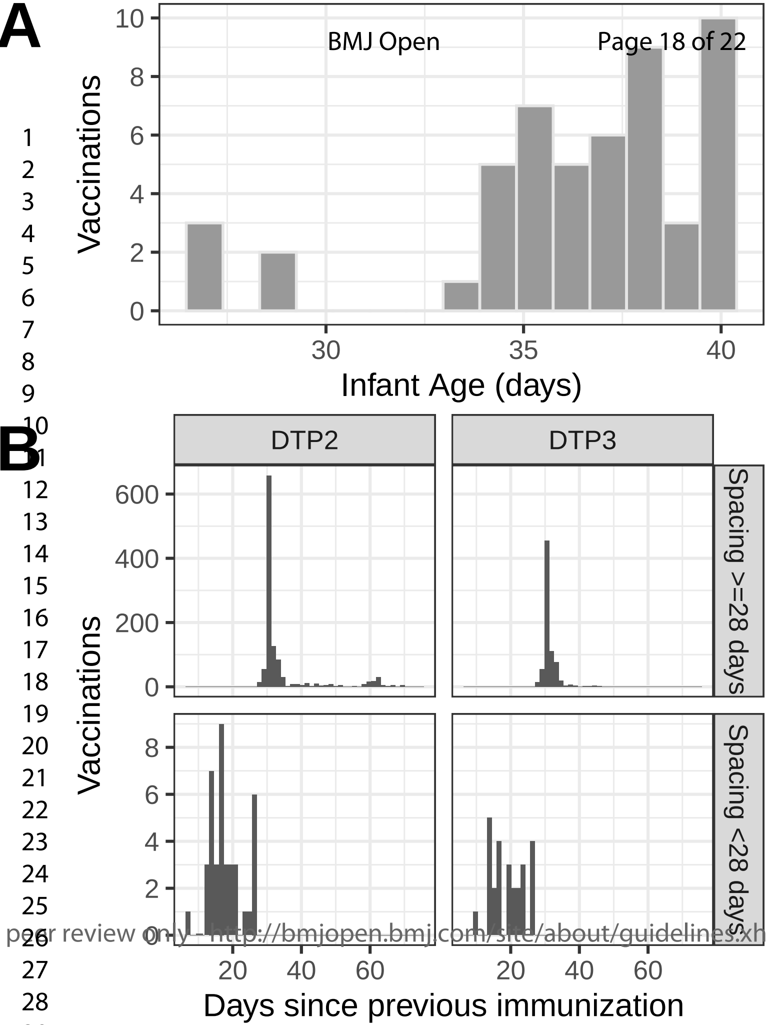
A
Vaccinations
10
8
6
4
2
0
30
35
40
Infant Age (days)
B
DTP2
DTP3
Spacing >=28 days
Spacing <28 days
600
400
200
0
8
6
4
2
0
20
40
60
Vaccinations
Days since previous immunization

STROBE Statement—Checklist of items that should be included in reports of ***cohort studies***

	Item No	Recommendation
**Title and abstract**	1	(*a*) Indicate the study's design with a commonly used term in the title or the abstract<br>**Title, "birth cohort"**
		(*b*) Provide in the abstract an informative and balanced summary of what was done and what was found<br>**Abstract, sections Methods and Results**
**Introduction**		
Background/rationale	2	Explain the scientific background and rationale for the investigation being reported<br>**Intro, Paras 1-3**
Objectives	3	State specific objectives, including any prespecified hypotheses<br>**Intro, Paras 3 & 5**
**Methods**		
Study design	4	Present key elements of study design early in the paper<br>**Methods, section Study Design**
Setting	5	Describe the setting, locations, and relevant dates, including periods of recruitment, exposure, follow-up, and data collection<br>**Methods, section Study Design**
Participants	6	(*a*) Give the eligibility criteria, and the sources and methods of selection of participants. Describe methods of follow-up<br>**Methods, section Study Design**
		(*b*) For matched studies, give matching criteria and number of exposed and unexposed<br>**NA**
Variables	7	Clearly define all outcomes, exposures, predictors, potential confounders, and effect modifiers. Give diagnostic criteria, if applicable<br>**Methods, section Assessment**
Data sources/ measurement	8*	For each variable of interest, give sources of data and details of methods of assessment (measurement). Describe comparability of assessment methods if there is more than one group<br>**Methods, section Assessment**
Bias	9	Describe any efforts to address potential sources of bias<br>**Methods, section Data and Statistical Analysis, last para**
Study size	10	Explain how the study size was arrived at<br>**Previously described (citation 18)**
Quantitative variables	11	Explain how quantitative variables were handled in the analyses. If applicable, describe which groupings were chosen and why<br>**Methods, section Data and Statistical Analysis**
Statistical methods	12	(*a*) Describe all statistical methods, including those used to control for confounding<br>**Methods, section Data and Statistical Analysis**
		(*b*) Describe any methods used to examine subgroups and interactions<br>**Methods, section Data and Statistical Analysis**
		(*c*) Explain how missing data were addressed<br>**Methods, section Data and Statistical Analysis**
		(*d*) If applicable, explain how loss to follow-up was addressed<br>**Methods, section Data and Statistical Analysis**
		(*e*) Describe any sensitivity analyses<br>**NA**

For peer review only - http://bmjopen.bmj.com/site/about/guidelines.xhtml

**Results**		
Participants	13*	(a) Report numbers of individuals at each stage of study—eg numbers potentially eligible, examined for eligibility, confirmed eligible, included in the study, completing follow-up, and analysed<br>**Results, Section Participation, Fig 1A, Table 1, Figure S1**
		(b) Give reasons for non-participation at each stage<br>**NA**
		(c) Consider use of a flow diagram<br>**Figure S1**
Descriptive data	14*	(a) Give characteristics of study participants (eg demographic, clinical, social) and information on exposures and potential confounders<br>**Results, Section Demographic Characteristics, Table 1, Figure 2**
		(b) Indicate number of participants with missing data for each variable of interest<br>**Figure 1A, Figure 3**
		(c) Summarise follow-up time (eg, average and total amount)<br>**Results, section Participation**
Outcome data	15*	Report numbers of outcome events or summary measures over time<br>**Figure 1 & 3, Tables 2-4**
Main results	16	(*a*) Give unadjusted estimates and, if applicable, confounder-adjusted estimates and their precision (eg, 95% confidence interval). Make clear which confounders were adjusted for and why they were included<br>**Figure 1 & 3, Tables 2-4**
		(*b*) Report category boundaries when continuous variables were categorized<br>**NA**
		(*c*) If relevant, consider translating estimates of relative risk into absolute risk for a meaningful time period<br>**NA**
Other analyses	17	Report other analyses done—eg analyses of subgroups and interactions, and sensitivity analyses<br>**Figure 4**
**Discussion**		
Key results	18	Summarise key results with reference to study objectives<br>**Discussion, paras 1-3**
Limitations	19	Discuss limitations of the study, taking into account sources of potential bias or imprecision. Discuss both direction and magnitude of any potential bias<br>**Discussion, paras 4-5**
Interpretation	20	Give a cautious overall interpretation of results considering objectives, limitations, multiplicity of analyses, results from similar studies, and other relevant evidence<br>**Discussion, paras 7-8**
Generalisability	21	Discuss the generalisability (external validity) of the study results<br>**Discussion, para 7,9**
**Other information**		
Funding	22	Give the source of funding and the role of the funders for the present study and, if applicable, for the original study on which the present article is based<br>**Section Funding**

*Give information separately for exposed and unexposed groups.

Note: An Explanation and Elaboration article discusses each checklist item and gives methodological background and published examples of transparent reporting. The STROBE checklist is best used in conjunction with this article (freely available on the Web sites of PLoS Medicine at http://www.plosmedicine.org/, Annals of Internal Medicine at http://www.annals.org/, and Epidemiology at http://www.epidem.com/). Information on the STROBE Initiative is available at http://www.strobe-statement.org.

For peer review only - http://bmjopen.bmj.com/site/about/guidelines.xhtml

Implementation and adherence of routine pertussis vaccination (DTP) in a low-resource urban birth cohort

Gunning et al.

2020

Supplemental Figures and Tables

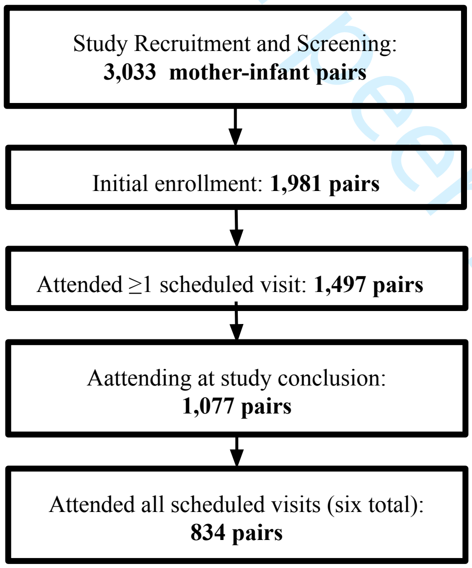

Figure S1: Study profile of birth cohort enrollment and attendance. Beyond eligibility and initial screening, the sole cause of cohort attrition was failure to attend one or more scheduled clinic visits.

For peer review only

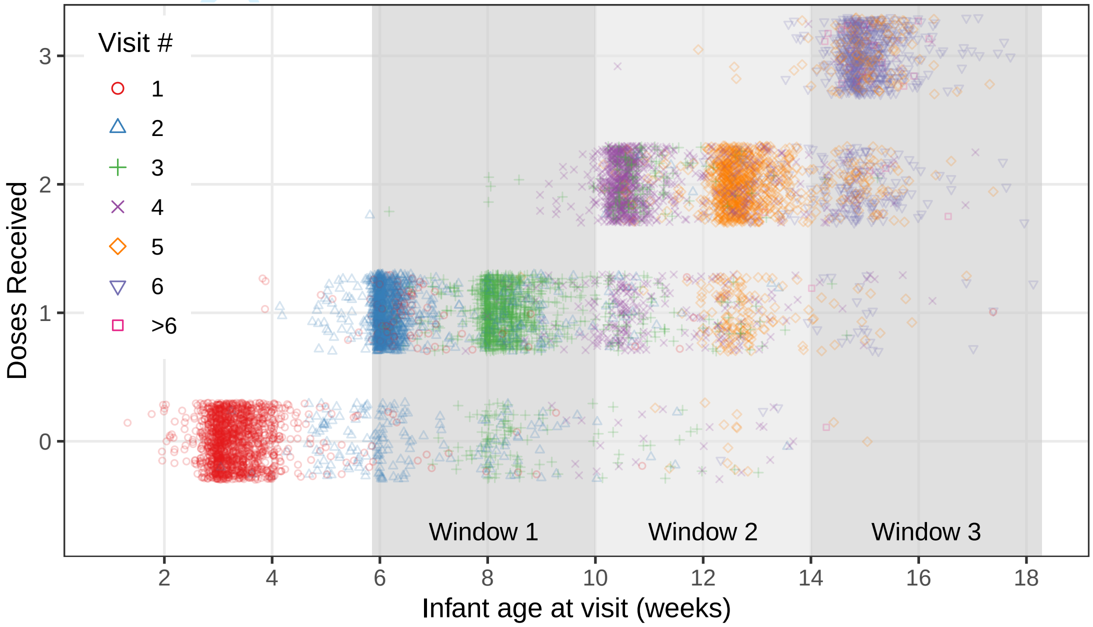

Figure S2: Infants were scheduled for 6 clinic visits over the course of the study. Figure shows the cumulative doses of DTP vaccine received per infant at each visit (color, shape) by infant age.

For peer review only - http://bmjopen.bmj.com/site/about/guidelines.xhtml

Implementation and adherence of routine pertussis vaccination (DTP) in a low-resource urban birth cohort

Journal:	*BMJ Open*
Manuscript ID	bmjopen-2020-041198.R1
Article Type:	Original research
Date Submitted by the Author:	16-Sep-2020
Complete List of Authors:	Gunning, Christian ; University of Georgia, Odum School of Ecology<br>Mwananyanda, Lawrence; Right to Care, Zambia<br>MacLeod, William; Boston University School of Public Health, Department of Global Health<br>Mwale, Magdalene; Right to Care, Zambia<br>Thea, Donald; Boston University School of Public Health, Department of Global Health<br>Pieciak, Rachel; Boston University School of Public Health, Department of Global Health<br>Rohani, Pejman; University of Georgia, Odum School of Ecology<br>Gill, Christopher; Boston University School of Public Health, Department of Global Health
**Primary Subject Heading**:	Infectious diseases
Secondary Subject Heading:	Public health, Paediatrics, Health informatics, Global health, Epidemiology
Keywords:	Public health < INFECTIOUS DISEASES, Community child health < PAEDIATRICS, Paediatric infectious disease & immunisation < PAEDIATRICS, Epidemiology < INFECTIOUS DISEASES

I, the Submitting Author has the right to grant and does grant on behalf of all authors of the Work (as defined in the below author licence), an exclusive licence and/or a non-exclusive licence for contributions from authors who are: i) UK Crown employees; ii) where BMJ has agreed a CC-BY licence shall apply, and/or iii) in accordance with the terms applicable for US Federal Government officers or employees acting as part of their official duties; on a worldwide, perpetual, irrevocable, royalty-free basis to BMJ Publishing Group Ltd ("BMJ") its licensees and where the relevant Journal is co-owned by BMJ to the co-owners of the Journal, to publish the Work in this journal and any other BMJ products and to exploit all rights, as set out in our licence.

The Submitting Author accepts and understands that any supply made under these terms is made by BMJ to the Submitting Author unless you are acting as an employee on behalf of your employer or a postgraduate student of an affiliated institution which is paying any applicable article publishing charge ("APC") for Open Access articles. Where the Submitting Author wishes to make the Work available on an Open Access basis (and intends to pay the relevant APC), the terms of reuse of such Open Access shall be governed by a Creative Commons licence – details of these licences and which Creative Commons licence will apply to this Work are set out in our licence referred to above.

Other than as permitted in any relevant BMJ Author's Self Archiving Policies, I confirm this Work has not been accepted for publication elsewhere, is not being considered for publication elsewhere and does not duplicate material already published. I confirm all authors consent to publication of this Work and authorise the granting of this licence.

For peer review only

Implementation and adherence of routine pertussis vaccination (DTP) in a low-resource urban birth cohort

April 1, 2020

Authors: CE Gunning*[1], L Mwananyanda[2], WB Macleod[3], M Mwale[2], DM Thea[3], RC Pieciak[3], P Rohani[1], and CJ Gill[3]

* Corresponding author (email: research@x14n.org)

[1] University of Georgia, Odum School of Ecology

[2] Right To Care Zambia

[3] Boston University School of Public Health, Department of Global Health

For peer review only

Abstract

Introduction

Reliable information on rates of up-to-date coverage and timely administration of routine childhood immunizations is critical for guiding public health efforts worldwide, yet prospective observation of vaccination programs within individual communities is rare. Here we provide a longitudinal analysis of the directly-observed administration of a 3-dose primary vaccination series to infants in a low-resource community in Lusaka, Zambia.

Methods

Throughout 2015, we recruited a longitudinal birth cohort of mother/infant pairs (initial enrollment, 1,981 pairs; attending, 1,497 pairs) from the peri-urban informal settlement of Chawama compound, located in Lusaka, Zambia. We prospectively monitored the administration of scheduled Diphtheria-Tetanus-Pertussis (DTP) vaccinations across the first 14-18 weeks of life. We analyzed study attendance and vaccine coverage, both overall and stratified by age group. We employed Kaplan-Meier analyses to estimate delays in age-appropriate administration of vaccine doses. We also assessed schedule timing violations, including early and compressed dose administration.

Results

At study completion, first dose (DTP1) rates were high (92.9% of attending), whereas third dose completion (DTP3) rates were far lower (61.9%). Missed vaccinations and study dropout both contributed to the low DTP3 completion rates. DTP1 was administered very late (at or after 10 weeks) to 61 infants (4.1%). DPT1 was administered too early to 64 infants (4.3%), and 77 (5.1%) received consecutive doses below the minimum recommended spacing of 28 days.

Conclusions

We observe substantial individual variation in the timing of early childhood DTP doses, though following this birth cohort proved challenging. Our results indicate that timely administration of both DTP1 and DPT3 remains a challenge in this community. These directly-observed, individual-based results provide an important counterpoint to more course-grained, survey-based national and province estimates of up-to-date vaccine coverage. This study also highlights the challenges of vaccine hesitancy and sub-optimal utilization of (no-cost) healthcare services in a low-resource urban setting.

Strengths and limitations of this study

- To our knowledge, this is the first prospective urban birth cohort study in a low-resource setting to directly document vaccinations at the place and time of administration.
- By following individuals across time, we are better able to assess healthcare utilization behavior relative to cross-sectional surveys.
- This study provides a detailed view of a single community across a year, and may not capture long-term trends or generalize to other populations.

Introduction

Estimates of vaccine coverage play a central role in epidemiology and public health, from identifying at-risk communities and age groups for supplementary immunization activities (SIAs) [1, 2] to their use in disease transmission models and subsequent inference [3–5]. Coverage estimates are also key indicators of national vaccination implementation success [6]. As such, high-quality vaccine coverage estimates are critical in shaping modern public health policy.

Estimates of childhood *up-to-date* vaccine coverage from cross-sectional studies (e.g., Demographic and Health Surveys (DHS) [7] and UNICEF [8]) have revealed numerous factors affecting vaccine administration in low- and middle-income countries (LMICs) [9–13]. However, these coarse-grained estimates can mask complexities in compliance and implementation. Particularly in LMICs, spatial and socioeconomic heterogeneities in coverage are common, as are delays in the timely administration of vaccine doses [9, 14]. Further, the reliability of surveys based on personal and informal records, i.e., vaccine cards and maternal recall, remains unclear [15].

With few exceptions [16, 17], detailed observations of childhood vaccinations during routine clinical care are lacking. Granular, community-based observations of individual patient histories can reveal temporal patterns in schedule adherence, including delays in age-appropriate vaccinations. Prospective observation of a cohort can also reveal if community-level DTP coverage differs substantially from regional coverage, and can help identify behavioral factors that affect the success of vaccine implementation programs, including vaccine hesitancy.

For peer review only - http://bmjopen.bmj.com/site/about/guidelines.xhtml

Here we provide a detailed analysis of infant vaccination in a sub-Saharan low-resource urban setting in Lusaka, Zambia. Throughout 2015, we followed a longitudinal birth cohort of mother-infant pairs from birth through 14 weeks of age as part of a broader study of pertussis transmission. Participants were scheduled for 6 clinic visits total; the primary 3-dose DTP series was administered at three of these visits according to the Zambian national schedule of 6, 10, and 14 weeks of age. Our team prospectively documented all clinic visits and administration of DTP doses using an electronic data capture system [18]. We quantified the DTP series dose trajectory of individual infants, and assessed the proportion of infants with up-to-date vaccinations, or who missed one or more doses. We evaluated compliance with published vaccine schedule guidelines, including minimum age restrictions and dose spacings.

In short, our use of directly-observed age-specific DTP administration data in a typical LMIC community clinic offers a unique window into vaccine implementation at the individual patient level that can help inform vaccine policy decisions globally.

Methods

Study design

The Southern Africa Mother Infant Pertussis Study (SAMIPS) was a longitudinal birth cohort study conducted in Lusaka, Zambia from March 2015-February 2016 to assess the incidence of pertussis in a representative population of otherwise healthy infants (details provided elsewhere [18]). Mother–infant pairs were enrolled from the population of the Chawama compound, a densely populated peri-urban slum of approximately 150,000 residents near central Lusaka. The study goal was to capture all live births that occurred in Chawama compound during the study period and, prior to study initiation, a public outreach campaign provided pregnant Chawama residents with information about the study. Infants were enrolled at the Chawama Primary Health Clinic (PHC) during their first scheduled postpartum well-child visit (at approximately 1 week old). The PHC is the only government-supported clinic in this community and, as such, is the primary source of medical care for Chawama residents. This feature allowed us to maximize study reach by capturing the majority of participant health care consumption in this community during the study period from a single point of service.

Enrollment eligibility required that infants were born after 37 weeks, weighed over 2500 grams, and were delivered without complications or apparent disease. Eligibility also required signed consent, Chawama residency (anticipated remaining in the community during study period), that the HIV status of mothers was known prior to delivery, and that HIV+ mothers were receiving prophylactic antiretroviral therapy at the time of delivery [18]. An extended discussion of study size is provided in [18].

Mothers were incentivized to join and remain in the cohort in three ways. First, the SAMIPS medical staff provided all routine and acute medical care for study participants during their time of enrollment. This significantly reduced clinic waiting times for care from over 3 hours to half an hour or less. Second, mothers received a travel stipend for each visit

For peer review only - http://bmjopen.bmj.com/site/about/guidelines.xhtml

valued at approximately 7 US dollars. Lastly, a small gift of baby supplies was provided for mothers attending the final scheduled study visit.

Enrolled infants were scheduled for six routine clinic visits at 2-3 week intervals through 14 weeks old (maximum 18 weeks). At each clinic visit, nasopharyngeal (NP) swabs were obtained from both mother and infant, and possible pertussis symptoms were recorded. Regular clinic staff provided routine childhood vaccinations at no cost. Administration of DTP doses 1-3 was scheduled at visits corresponding to 6, 10, and 14 weeks of age according to Zambian national guidelines (see WHO Expanded Programme of Immunization (EPI) schedule [19]). DTP vaccinations were administered as a pentavalent combination (Pentavac, Serum Institute of India Limited, Pune, India) that included whole-cell pertussis, *Haemophilus influenzae* type B (HIB), and Hepatitis B. The pneumococcal conjugate vaccine (PCV10) was co-administered with DTP; oral rotavirus vaccine administration was scheduled for 6 and 10 weeks of age.

At each visit, infant vaccination cards ("under 5 cards") were reviewed by clinic staff, and the dates of any previously unrecorded vaccinations were noted. When participants missed a scheduled visit, clinic staff attempted to contact participants by phone to reschedule the visit.

Patient and Public Involvement

This research was done without patient involvement. Patients were not invited to comment on the study design and were not consulted to develop patient relevant outcomes or interpret the results. Patients were not invited to contribute to the writing or editing of this document for readability or accuracy.

Assessment of Vaccine Administration

For protection against pertussis, the WHO recommends that infants receive a 3-dose primary series with a DTP-containing vaccine [19]. Guidelines specify a minimum administration age of 6 weeks for the first dose (DTP1), a minimum interval of 4 weeks between subsequent doses, and a target inter-dose interval of 4-8 weeks. In Zambia, pregnant mothers receive one or more doses of tetanus toxoid monovalent vaccine during pregnancy.

For this analysis, we use the Zambian 6-10-14 week EPI schedule for DTP to define target age windows in which infants should have received each DTP dose: window 1, 41-69 days; window 2, 70-97 days; window 3, 98-126 days. Note that window 1 extends one day early to account for uncertainty the exact timing birth and vaccination. We tabulate the number of doses each infant received in each window, and at study completion. For example, an infant might receive no doses during window 1, and a dose in both windows 2 and 3, for a total of two doses at study completion.

Infants who received all scheduled doses to-date were categorized as *up-to-date*. However, once a window was missed, an infant was no longer up-to-date during subsequent age windows. Infants who received no doses by the end of the dose window were categorized

For peer review only - http://bmjopen.bmj.com/site/about/guidelines.xhtml

as *unvaccinated*. Vaccinations that occurred >1 day prior to window 1 were classified as *early*. Vaccinations administered less than 4 weeks after a preceding dose were classified as *compressed*.

Data and Statistical Analyses

Administration of all DTP doses by clinic staff during the study period was directly observed and documented by study staff. All data were manually recorded on standardized data sheets, and digitized using a digital pen electronic data capture system (XCallibre, Durban, South Africa. https://www.xcallibre.com). Data were de-identified prior to analysis: each mother-infant pair was assigned a random date offset of +/-3 days, and all study dates (i.e., birth and visits) were adjusted by this offset.

Mother-infant pairs were considered *attending* (on-study) from enrollment until their last recorded clinic visit during the study period. We exclude from further consideration individuals who enrolled in SAMIPS but did not attend any scheduled clinic visits.

We report the number of infants who received exactly (and at least) 0-3 doses by study completion, along with the percent of attending study infants in each of these groups.

We stratified dose outcomes by age window. For each window, we report the number of *unique* infants attending at least one scheduled visit. We also report the number of infants who: received or missed a vaccine dose, received compressed or early vaccinations, and were up-to-date (along with respective percentages). Since attrition and irregular attendance are expected in cohort studies, we compute percentages relative to the number of unique infants in each window (above).

We perform a modified Kaplan-Meier analysis of vaccination delays [20, 14]. The standard Kaplan-Meier method assumes that non-attending individuals (i.e., right-censored) experience events at the same rate as attending individuals. Here we explore an alternative worst-case scenario where non-attending individuals receive no further vaccinations during the study period (subsequent to study drop-out). While subsequent make-up vaccinations may occur, our assumption of minimal off-study vaccination is plausible, as the Chawama clinic is the sole provider of DTP in this community, and study staff recorded all DTP doses there during the study period.

Results

Participation

Infants were enrolled in the study between March and December 2015 (1,981 out of 3,033 screened). The five most common reasons for study exclusion include lack of consent (14.3%), low infant birth weight (7.9%), community non-residence (5.9%), mother anticipates moving (4.1%), and mother not 18-39 years old (3.7%) (see Supplemental Table 3 in [18] for enrollment details). Enrollees that did not attend any subsequent clinic visits (484 total) were removed from this analysis, leaving 1,497 initially attending infants. We depict study participation over time of the remaining infants in Figure 1A, while Figures 1B-

For peer review only - http://bmjopen.bmj.com/site/about/guidelines.xhtml

C detail the timing of study events. At the beginning of windows 1-3, 1,400 infants (window 1), 1,306 infants (window 2), and 1,089 infants (window 3) were still attending (see Figure S1 for study profile). The observed drop-off in cohort size near the end of window 3 represents graduation from the study, as infants' final clinic visits (and DTP dose) were scheduled for approximately 14 weeks of age. Overall, the duration of study attendance was a median of 96 days (min=12d, max=120d).

Demographic Characteristics

Overall, the demographic characteristics of participants did not co-vary with duration of study attendance (see Table 1), including all enrolled individuals, individuals attending at least one scheduled visits (attending), and individuals attending all scheduled visits (completed). Mothers' median age was 25 years, regardless of attendance. Across enrolled mothers, 17.5% were HIV+, while slightly more attending mothers were HIV+ (19.3%). Infants' median birth weight was 3000 grams. Regardless of attendance, median household size was 1 infant of <1 year of age (including study infant) and 2 children of <5 years of age (including infants).

With one exception, demographic characteristics did not affect the relative risk of the following end-of-study outcomes: being unvaccinated, receiving at least one dose (DTP1), and receiving all three doses (DTP3) (Figure 2). We did observe a marginally lower risk of DTP3 (i.e., higher risk of incomplete vaccination) in infants who received OPV at birth (RR=0.89, 95% CI: 0.81-0.97).

Table 1: **Demographic characteristics of participants** by duration of study attendance (columns), showing percentage or median, as appropriate. Interquartile range is shown in parentheses. **Attending** mother-infant pairs attended one or more scheduled clinic visit. **Completed** individuals attended all six scheduled visits.

	Duration of		
Parameter	Enrolled	Attending	Completed
Number Under Study	1981	1497	834
**Mothers**			
Married	90.2%	90.1%	90.3%
HIV+	17.5%	19.3%	18.6%
Median Age	25 (21, 29)	25 (22, 29)	25 (22, 30)
Median Infants In House (<1 year)	1 (1, 1)	1 (1, 1)	1 (1, 1)
Median Children In House (< 5 years)	2 (1, 2)	2 (1, 2)	2 (1, 2)
**Infants**			
Born at Chawama PHC	56.9%	56.7%	58.2%
Born at UTH	34.8%	35.5%	34.3%
Female Sex	46.9%	46.4%	46.4%
Received BCG at birth	46.4%	46.6%	46.2%
Received OPV at birth	33.4%	32.4%	27.7%
Median Birth Weight (kg)	3 (2.8, 3.3)	3 (2.8, 3.3)	3.1 (2.8, 3.3)

For peer review only - http://bmjopen.bmj.com/site/about/guidelines.xhtml

Scheduled Doses

Vaccination outcomes at the completion of the study (total doses received) are summarized in Table 2, while Table 3 details the cumulative doses received at the end of each age window. Overall, approximately 7.1% (107/1,497) of attending infants had not received any DTP vaccinations at the conclusion of the study (Table 2). This corresponds to 9.8% (107/1,089) of infants who remained in the cohort up to the beginning of window 3 (and thus had the opportunity to receive each of the three scheduled doses, see Figure 1A).

The timing of DTP dose administration to individual participants is shown in Figure 1B (for a detailed view of all clinic visits, see Figure S2). We also aggregate these data by week, highlighting that most infants received each DTP dose within a week of the target schedule of 6, 10, and 14 weeks of age (Figure 1C). However, deviations from the planned schedule were common. For example, early DTP vaccinations were administered to 64 infants prior to window 1 (Table 3), before which no vaccinations should have occurred (except for doses of the oral polio and BCG vaccines administered at birth, see Table 1). Similarly, the spread of events to the right of the target administration age of 6, 10 and 14 weeks represents vaccination after the scheduled delivery date (Fig 1B-1C). Two general trends here are apparent across the three scheduled doses. With each subsequent dose: 1), the average time of administration drifts farther to the right of (later than) the target schedule; and 2) the clustering of administration times becomes more dispersed.

We also observed infants who missed a dose and later received a subsequent dose on (or near) schedule (Figures 1B-C). For example, 118 infants received their first DTP dose during window 2 , while 170 infants received a second DTP dose during window 3 (Table 3, Figure 1C). Since the study did not follow participants beyond age 16 weeks (with few exceptions), it remains unclear whether (and when) catch-up of any doses missed during the study might have occurred.

Time-stratified results based on infant visits within each age window are shown in Table 4. These results are less sensitive to cohort attrition, as they assess outcomes over a shorter time period. Here, we find that up-to-date rates fell across the study from 95.8% of visiting infants (window 1, DTP1) to 88.4% (window 2, DTP2) and finally to 83.7% (window 3, DTP3). The unvaccinated rate of visiting infants also fell from window 1 (12.3%) to window 2 (8.8%), but did not decrease further during window 3 (9.9%). We note that many infants who visited the clinic in windows 1-3 nonetheless missed scheduled vaccinations:6.6% (window 1), 9.1% (window 2), and 8.4% window 3.

Table 2: **Vaccine status at study completion.** Number of infants that received at least (or exactly) 0-3 doses of DTP at study completion. Percentages are rounded (out of 1,497 attending infants).

N	Exactly N Doses (%)	At Least N Doses (%)
**0**	107 (7.1)	1497 (100.0)

**1**	147 (9.8)	1390 (92.9)
**2**	316 (21.1)	1243 (83.0)
**3**	927 (61.9)	927 (61.9)

Table 3: **Cumulative doses received across study.** Number of unique infants that received DTP1-3 by the *end* of each age window (columns, in days). Bottom row shows total doses administered in each window (some infants received more than one dose per window).

	**Early**	**Window 1**	**Window 2**	**Window 3**
**Received**	**[0,41)**	**[41,70)**	**[70,98)**	**[98,128)**
**DTP1**	64	1264	55	6
**DTP2**		36	1117	90
**DTP3**			20	907
**Any**	64	1300	1192	1003

For peer review only

Table 4: **Vaccinations stratified by age window,** showing number of unique infants *within* each age window. **Visits:** infants attending ≥1 scheduled visit. **Received, Missed:** infants that received (or did not receive) a DTP dose. **Compressed:** infants vaccinated <4 weeks after previous dose (see also Fig. 4B). **Up-to-date:** infants that received the targeted N*th* dose in this window. **Unvaccinated:** infants that received zero total doses by end of window. Percentages are rounded (out of **Visits**).

Age Window	Target (N)	Visits	Received (%)	Missed (%)	Compressed (%)	Up-to-date (%)	Unvaccinated (%)
**[41,70)**	**1**	1378	1287 (93.4)	91 (6.6)	-	1320 (95.8)	169 (12.3)
**[70,98)**	**2**	1301	1183 (90.9)	118 (9.1)	50 (3.8)	1150 (88.4)	114 (8.8)
**[98,128)**	**3**	1093	1001 (91.6)	92 (8.4)	47 (4.3)	915 (83.7)	108 (9.9)

Vaccine timing

A timely initial dose plays an important role in protecting infants from preventable morbidity and mortality [21]; prior to DTP1, study infants' protection against pertussis depended on unknown but presumably low and waning maternally-derived immunity. We observed 61

infants (4.1% of attending) that received DTP1 at 10 weeks of age or greater, though only 6 (0.4% of attending) received DTP1 at 14 weeks of age or greater (Table 3, Figure 3).

The Kaplan-Meier survival analysis (assuming no vaccination in infants lost to follow-up) revealed a modest median delay in the timely administration of DTP1 (1 day), though the median delay grew across primary series to 5 days (DTP2) and 9 days (DTP3). However, many infants experienced more substantial delays: for DTP1 the upper decile delay (slowest 10%) was 32 days, and for DTP2 the upper quantile delay (slowest 25%) was 17 days. Finally, 570 infants (38.1% of attending) did not receive DTP3 during the study.

By comparison, schedule violations involving early and compressed doses were minor. As noted above, DTP1 was administered too early to 64 infants (4.3% of attending), with most of these doses administered 2-7 days early (at age 35-40 days) (Table 3, Figure 4A). The median spacing between subsequent doses (31 days) slightly exceeded the minimum recommended spacing of 28 days (Figure 4B). A small minority of infants experienced dose compression between doses 1-2 and/or doses 2-3: 77 infants received doses spaced less than 25 days apart, while 48 infants received doses less than 20 days apart.

Discussion

This study offers a descriptive analysis of prospectively-collected, carefully documented observations of DTP vaccinations administered in an urban African public health clinic. Overall, we found that 61.9% of attending infants (927/1,497) received the full DTP3 series in a timely manner, corresponding to 85.1% of infants who attended into window 3 (and were thus available for a third dose, 791/1,089). . This is remarkable given that Chawama's EPI implementation lacks direct client outreach or other supportive interventions. However, we were concerned by higher than anticipated rate of missed (and delayed) doses: at study completion, 9.8% of attending infants had received only one dose, while 7.1% of attending infants lacked records for any DTP vaccinations. In short, our findings suggest that access to (no-cost) healthcare, available to all study infants, was not the sole limiting factor in DTP uptake in Chawama compound.

While not directly comparable with our results, WHO-UNICEF estimated that Zambia's 2015 up-to-date DTP3 coverage was 90% [22], much higher than our findings of 61.9%. However, our DTP1 rates at study completion (92.9%) were proportionally closer to corresponding WHO-UNICEF estimates (97%) and 2013-2014 Zambia DHS estimates for Lusaka province (97.8%) [23] (though the ceiling effect leaves little range for observable differences here). Overall, our results suggest that delays in timely vaccinationwarrants further attention in Zambia, as does spatial heterogeneity (e.g., across socioeconomic gradients).

The longitudinal nature of this study allowed us to resolve key implementation features that cross-sectional or survey-based approaches cannot resolve, including dose timing. In this cohort, early and compressed vaccinations were relatively rare. However, we observed that administration of scheduled vaccinations was more variable and, on average, less timely with each subsequent dose. This pattern indicates that delays are not random across life, and suggests that delays early in life have down-stream effects on the timing of subsequent

For peer review only - http://bmjopen.bmj.com/site/about/guidelines.xhtml

vaccinations. We also gained insights into how individuals' behavior affected population-level coverage rates. For example, the small change in unvaccinated infants attending window 2 (114, 8.8%) and window 3 (108, 9.9%) suggests that doses are not skipped randomly by study participants. Rather, a core group of individuals appears to have regularly attend clinic visits while opting out of (no-cost) routine vaccines, despite encouragement by our study team. Indeed, earlier qualitative work in this cohort suggests that vaccine hesitancy plays a non-trivial role here

[24].

Following a birth cohort across almost five months of life proved challenging in this setting. We experienced high rates of initial non-participation among eligible infants (484 out of 1,981 initial enrollees), as well as ongoing attrition of the cohort throughout the study. Indeed, only 72.7% of attending infants (1,089, Figure 1) participated in the study up to an age of 14 weeks (98 days), and were thus available to receive all scheduled DTP doses during the study. However, our intensive prospective monitoring of the primary source of health care in Chawama leads us to believe that enrollees who missed one or more visits rarely receive unrecorded vaccinations at the PHC during the study period. In addition, we expect that non-clinic vaccinations of enrollees during this period were rare due to cost and geographical access. On the other hand, study involvement could have introduced an upwards bias on clinic visit behavior and DTP uptake relative to this population's background, non-study health care behavior.

Two unresolved questions remain: 1) whether infants who left the cohort early were subsequently vaccinated at the PHC after study graduation or were vaccinated elsewhere off-study, and 2) whether attending infants who missed scheduled doses later received catch-up doses, e.g., at subsequent well-child visits. In Lusaka, catch-up of missed doses is available at monthly clinic visits and two targeted "child health weeks" (June and November). However, these supplemental vaccination campaigns are implemented without respect to known vaccination compliance (as documented in each child's under five card), and thus do not systematically target children who missed doses during the primary series. Moreover, after the final study visit (at age 14-16 weeks), the next routine well child visit is not until nine months of age, when the measles vaccine dose is given. As such, even when supplemental vaccination occurs, the subsequent delays in timely vaccination may be substantial.

We believe this study fills an important gap in the childhood vaccination literature. As a periurban informal settlement, Chawama represents a typical under-served community in a LMIC, where a single public clinic is the primary health care resource for more than 150,000 residents. This study provides an important point of comparison for additional such prospective studies, in order to better assess variation by geographic region or resource availability. We also believe that further investigation of these findings could provide significant value to the public health community. For example, while beyond the scope of this study, a cross-sectional seroconversion survey of young children (e.g., 1-5 years of age) could help quantify the frequency of late or absent vaccination stratified by age. For example,

For peer review only - http://bmjopen.bmj.com/site/about/guidelines.xhtml

seropositivity for tetanus antibodies could provide a robust indicator of (at least) DTP1 uptake, as tetanus toxoid is highly immunogenic and elicits durable immunity [25].

Despite lingering uncertainties, our analyses raise broad questions about pertussis control in low-resource settings, including herd immunity and epidemic preparedness. Crowded living situations can facilitate pertussis transmission relative to less dense regions [26, 27], while the scarcity of macrolide treatments in this community may yield longer-lasting infections [28]; both effects could raise the critical vaccination threshold required for herd immunity [29]. Further, lower DTP coverage here relative to Lusaka province is consistent with the previously-described impacts of socioeconomic status [10–13]. Together, these forces could allow dense informal settlements to serve as foci for more widespread pertussis epidemics. Our results also highlight the potential public health consequences of such an epidemic. We directly observed 169 infants (11.3% of attending) who remained unvaccinated at age 10 weeks, along with a corresponding 114 infants (7.6%) at age 14 weeks. These infants were at elevated risk of severe disease, hospitalization, and death [30, 31], yet low-resource settings such as Chawama are ill-prepared to treat severe cases or high case loads. Indeed, the PHC has only 10 hospital beds to serve this community.

These results have additional implications for the full package of vaccines administered via the EPI schedule, as failure to receive a scheduled DTP dose may also indicate failure to receive additionally scheduled vaccinations. For example, both the pneumococcal conjugate and rotavirus vaccines are commonly co-administered with DTP. We expect that marginal improvements in EPI implementation would be highly impactful to public health outcomes, particularly in under-served communities, and that detailed descriptions of when and why EPI schedule violations occur will help identify and remediate existing barriers to vaccine uptake in these communities.

In summary, this study provides a uniquely granular view of routine DTP implementation in a low-resource urban African setting that highlights individual behavior. Our data suggest that delayed age of first vaccination, along with possible vaccine hesitancy and loss of herd immunity, poses a significant public health risk to this community, echoing calls to strengthen EPI programs in low-resource urban settings [32, 33].

Competing Interests

The authors declare no competing interests.

Data Sharing

De-identified data will be made available in a public, open access repository (Data Dryad) prior to publication. The R code used to produce all analyses and figures is available on request.

Author Contributions

Conceived study: CJG. Designed study: CJG, CEG. Conducted analysis, created figures and tables: CEG. Contributed to and interpreted analysis: CEG, WBM, PR, CJG. Wrote manuscript: CEG, PR, CJG. Contributed to manuscript revisions: CEG, LM, WBM, MM, DMT, RCP, PR, CJG.

Funding

The Bill & Melinda Gates Foundation funded the initial SAMIPS cohort (OPP 1105094). The National Institutes of Health/National Institute of Allergy and Infectious Diseases supported all subsequent analyses, including this analysis, using the SAMIPS database (1R01 AI133080). The study sponsors had no role in study design, analysis, or publication.

For peer review only - http://bmjopen.bmj.com/site/about/guidelines.xhtml

References

[1] Marita Zimmermann, Kurt Frey, Brittany Hagedorn, AJ Oteri, Abdulazeez Yahya, Maimuna Hamisu, Fred Mogekwu, Faisal Shuaib, Kevin A McCarthy, and Guillaume Chabot-Couture. Optimization of frequency and targeting of measles supplemental immunization activities in Nigeria: A cost-effectiveness analysis. *Vaccine*, 37 (41):6039–6047, 2019.

[2] Maria Joyce U Ducusin, Maricel de Quiroz-Castro, Sigrun Roesel, Luzviminda C Garcia, Dulce Cecilio-Elfa, W William Schluter, James L Goodson, and Eugene Lam. Using the World Health Organization measles programmatic risk assessment tool for monitoring of supplemental immunization activities in the Philippines. *Risk Analysis*, 37(6):1082–1095, 2017.

[3] Manoj Gambhir, Thomas A Clark, Simon Cauchemez, Sara Y Tartof, David L Swerdlow, and Neil M Ferguson. A change in vaccine efficacy and duration of protection explains recent rises in pertussis incidence in the United States. *PLoS Comput Biol*, 11(4):e1004138, 2015.

[4] Yoon Hong Choi, Helen Campbell, Gayatri Amirthalingam, Albert Jan Van Hoek, and Elizabeth Miller. Investigating the pertussis resurgence in England and Wales, and options for future control. *BMC medicine*, 14(121): 1–11, 2016.

[5] Matthieu Domenech de Cell`es, Felicia M. G. Magpantay, Aaron A. King, and Pejman Rohani. The impact of past vaccination coverage and immunity on pertussis resurgence. *Science Translational Medicine*, 10(434), 2019. ISSN 1946-6234. doi: 10.1126/scitranslmed.aaj1748. URL https://stm.sciencemag.org/content/10/434/eaaj1748.

[6] Jean-Marie Okwo-Bele and Thomas Cherian. The Expanded Programme on Immunization: a lasting legacy of smallpox eradication. *Vaccine*, 29:D74–D79, 2011.

[7] Madeleine Short Fabic, YoonJoung Choi, and Sandra Bird. A systematic review of demographic and health surveys: data availability and utilization for research. *Bulletin of the World Health Organization*, 90:604–612, 2012.

[8] Anthony Burton, Roeland Monasch, Barbara Lautenbach, Marta Gacic-Dobo, Maryanne Neill, Rouslan Karimov, Lara Wolfson, Gareth Jones, and Maureen Birmingham. WHO and UNICEF estimates of national infant immunization coverage: methods and processes. *Bulletin of the World Health Organization*, 87:535–541, 2009. URL https://www.who.int/bulletin/volumes/87/7/08-053819/en/.

[9] Andrew Clark and Colin Sanderson. Timing of children's vaccinations in 45 low-income and middle-income countries: an analysis of survey data. *The Lancet*, 373(9674):1543–1549, 2009.

[10] Mary-Christine Sullivan, Ayalew Tegegn, Fasil Tessema, Sandro Galea, and Craig Hadley. Minding the immunization gap: family characteristics associated with completion rates in rural Ethiopia. *Journal of Community Health*, 35(1):53–59, 2010.

[11] Ahmad Reza Hosseinpoor, Nicole Bergen, Anne Schlotheuber, Marta Gacic-Dobo, Peter M Hansen, Kamel Senouci, Ties Boerma, and Aluisio JD Barros. State of inequality in Diphtheria-Tetanus-Pertussis immunisation coverage in low-income and middle-income countries: a multi-country study of household health surveys. *The Lancet Global Health*, 4(9): e617–e626, 2016.

[12] Jin-Won Noh, Young-mi Kim, Nabeel Akram, Ki-Bong Yoo, Jumin Park, Jooyoung Cheon, Young Dae Kwon, and Jelle Stekelenburg. Factors affecting complete and timely childhood immunization coverage in Sindh, Pakistan; a secondary analysis of cross-sectional survey data. *PloS one*, 13(10):e0206766, 2018.

[13] Ikuma Nozaki, Masahiko Hachiya, and Tomomi Kitamura. Factors influencing basic vaccination coverage in Myanmar: secondary analysis of 2015 Myanmar demographic and health survey data. *BMC public health*, 19(1): 242, 2019.

[14] Manas K Akmatov and Rafael T Mikolajczyk. Timeliness of childhood vaccinations in 31 low and middle-income countries. *J Epidemiol Community Health*, 66(7):e14–e14, 2012.

[15] Melody Miles, Tove K Ryman, Vance Dietz, Elizabeth Zell, and Elizabeth T Luman. Validity of vaccination cards and parental recall to estimate vaccination coverage: a systematic review of the literature. *Vaccine*, 31 (12):1560–1568, 2013.

[16] Shankar Prinja, Madhu Gupta, Amarjeet Singh, and Rajesh Kumar. Effectiveness of planning and management interventions for improving age-appropriate immunization in rural India. *Bulletin of the World Health Organization*, 88:97–103, 2010. doi: 10.2471/BLT.08.059543.

For peer review only

[17] Ayebo Evawere Sadoh, Damian Uchechukwu Nwaneri, Bamidele Charity Ogboghodo, and Wilson Ehidiamen Sadoh. Effect of introduction of pentavalent vaccine as replacement for Diphtheria–Tetanus–Pertussis and Hepatitis B vaccines on vaccination uptake in a health facility in Nigeria. *Vaccine*, 34(24):2722–2728, 2016.

[18] Christopher J Gill, Lawrence Mwananyanda, William MacLeod, Geoffrey Kwenda, Magdalene Mwale, Anna L Williams, Kazungu Siazeele, Zhaoyan Yang, James Mwansa, and Donald M Thea. Incidence of severe and non-severe pertussis among HIV-exposed and-unexposed Zambian infants through 14 weeks of age: results from the southern Africa mother infant pertussis study (SAMIPS), a longitudinal birth cohort study. *Clinical Infectious Diseases*, 63(suppl 4):S154–S164, 2016.

[19] World Health Organization. Pertussis vaccines: WHO position paper—august 2015. *Weekly Epidemiological Record*, 90(35):433–460, 2015.

[20] B Laubereau, M Hermann, HJ Schmitt, J Weil, and R von Kries. Detection of delayed vaccinations: a new approach to visualize vaccine uptake. *Epidemiology & Infection*, 128(2):185–192, 2002.

[21] Zhiyun Chen and Qiushui He. Immune persistence after pertussis vaccination. *Human vaccines & immunotherapeutics*, 13(4):744–756, 2017.

[22] WHO. WHO-UNICEF estimates time series for Zambia (ZMB). http://apps.who.int/immunization_monitoring/globalsummary/estimates?c=ZMB. Accessed: 2019-10-01.

[23] Central Statistical Office/Zambia, Ministry of Health/Zambia, University of Zambia Teaching Hospital Virology Laboratory, University of Zambia Department of Population Studies, Tropical Diseases Research Centre/Zambia, and ICF International. Zambia demographic and health survey 2013-14. http://dhsprogram.com/ pubs/pdf/FR304/FR304.pdf, 2015.

[24] Anna Larson Williams, Rachel Mitrovich, Lawrence Mwananyanda, and Chris Gill. Maternal vaccine knowledge in low-and middle-income countries—and why it matters. *Human vaccines & immunotherapeutics*, 15(2):283– 286, 2019.

[25] Yanping Li, Rong Cheng Li, Qiang Ye, Changgui Li, You Ping Liu, Xiao Ma, Yanan Li, Hong Zhao, Xiaoling Chen, Deepak Assudani, et al. Safety, immunogenicity and persistence of immune response to the combined Diphtheria, Tetanus, acellular Pertussis, poliovirus and Haemophilus influenzae type b conjugate vaccine (dtpaipv/hib) administered in Chinese infants. *Human vaccines & immunotherapeutics*, 13(3):588–598, 2017.

[26] Pamela Rendi-Wagner, Joshua Tobias, Larisa Moerman, Sophy Goren, Ravit Bassal, Manfred Green, and Dani Cohen. The seroepidemiology of Bordetella pertussis in Israel—estimate of incidence of infection. *Vaccine*, 28 (19):3285–3290, 2010.

[27] FMG Magpantay and P Rohani. Dynamics of pertussis transmission in the United States. *American journal of epidemiology*, 181(12):921–931, 2015.

[28] Centers for Disease Control and Prevention. Recommended antimicrobial agents for the treatment and postexposure prophylaxis of pertussis: 2005 CDC guidelines. *MMWR*, 54(RR-14):1–16, 2005.

[29] R.M. Anderson and R.M. May. *Infectious diseases of humans*. Oxford University Press Oxford, 1991.

[30] Veronica Kolos, Robert Menzies, and Peter McIntyre. Higher pertussis hospitalization rates in indigenous Australian infants, and delayed vaccination. *Vaccine*, 25(4):588–590, 2007.

[31] Helen Campbell, Gayatri Amirthalingam, Nick Andrews, Norman K Fry, Robert C George, Timothy G Harrison, and Elizabeth Miller. Accelerating control of pertussis in England and Wales. *Emerging infectious diseases*, 18 (1):38, 2012.

[32] Md Jasim Uddin, Charles P Larson, Elizabeth Oliveras, AI Khan, MA Quaiyum, and Nirod Chandra Saha. Child immunization coverage in urban slums of Bangladesh: impact of an intervention package. *Health policy and planning*, 25(1):50–60, 2009.

[33] Martin K Mutua, Elizabeth Kimani-Murage, and Remare R Ettarh. Childhood vaccination in informal urban settlements in Nairobi, Kenya: who gets vaccinated? *BMC public health*, 11(1):6, 2011.

For peer review only

Figure Captions

Figure 1. **A:** Study participation by infant age. Shaded regions show scheduled administration of DTP doses 1-3. **B:** Timeline of DTP dose administration (see also Figure S2). C: Number of vaccinations per week, by dose number.

Figure 2. Relative risk of vaccination outcome (columns) by demographic covariate (y-axis), showing 95% CI. Select p-values are shown above estimates. Covariates: @birth indicates the respective vaccine was administered at birth; HIV+ & ART indicates the mother was HIV+ and taking antiretroviral therapy at time of enrollment. See Table 2 for outcome frequencies.

Figure 3. Kaplan-Meier analysis: estimated dose administration probability for DTP doses 1-3, assuming no vaccination in infants lost to follow-up. Vertical grey lines show dose target age; intersections with horizontal dashed lines show the median, upper quantile (slowest 25%) and upper decile (slowest 10%) delays for each dose. Tables show number of infants at select ages.

Figure 4. **A:** Number of early vaccinations (DTP1) by infant age. **B:** Number of vaccinations by dose spacing (time since previous dose), split by dose number (columns). Rows show compressed versus appropriate spacing (rows).

For peer review only

For peer review only - http://bmjopen.bmj.com/site/about/guidelines.xhtml

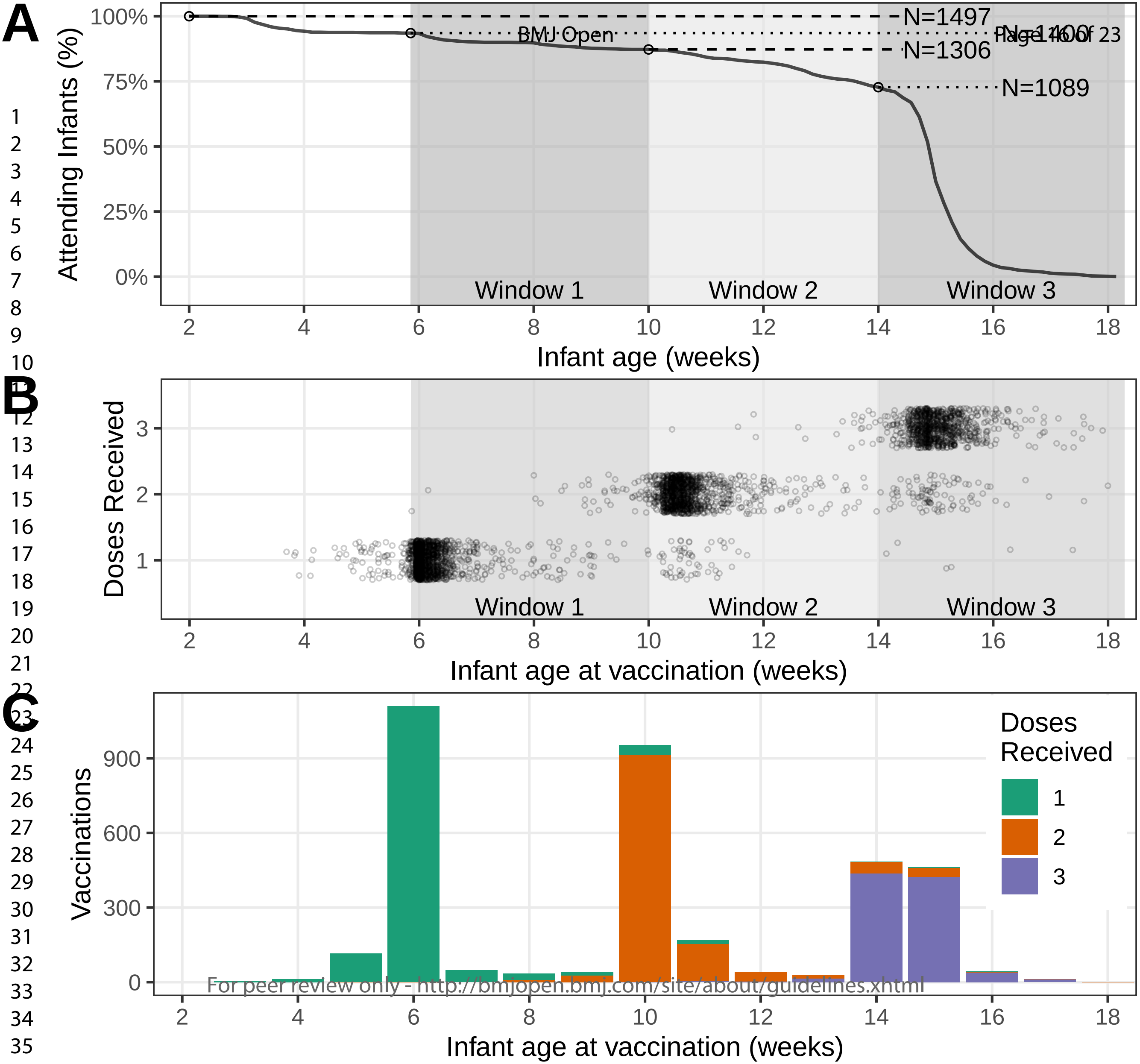

For peer review only - http://bmjopen.bmj.com/site/about/guidelines.xhtml

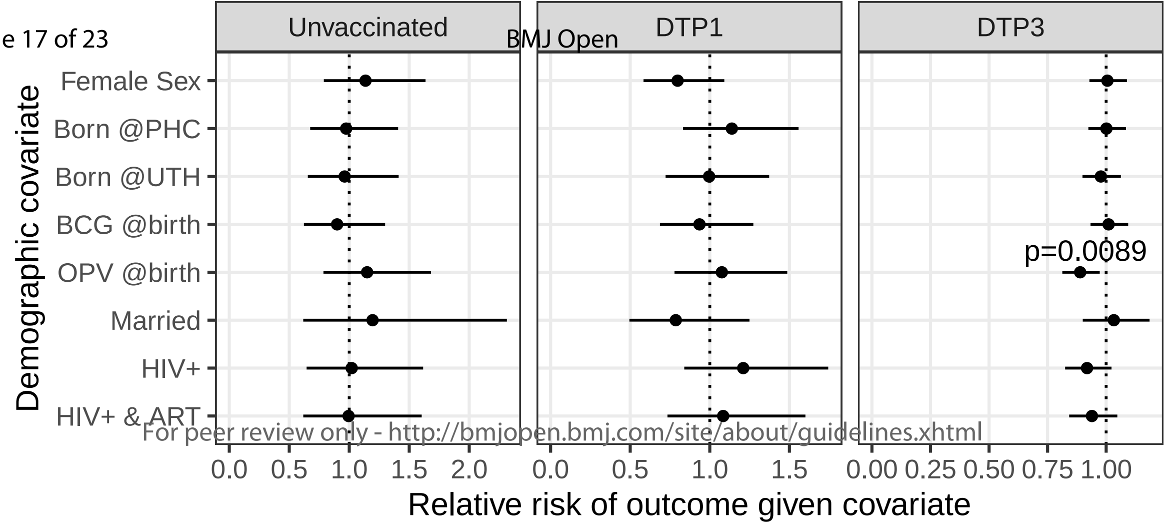
Unvaccinated
DTP1
DTP3
Demographic covariate
Female Sex
Born @PHC
Born @UTH
BCG @birth
OPV @birth
Married
HIV+
HIV+ & ART
p=0.0089
0.0 0.5 1.0 1.5 2.0
0.0 0.5 1.0 1.5
0.00 0.25 0.50 0.75 1.00
Relative risk of outcome given covariate

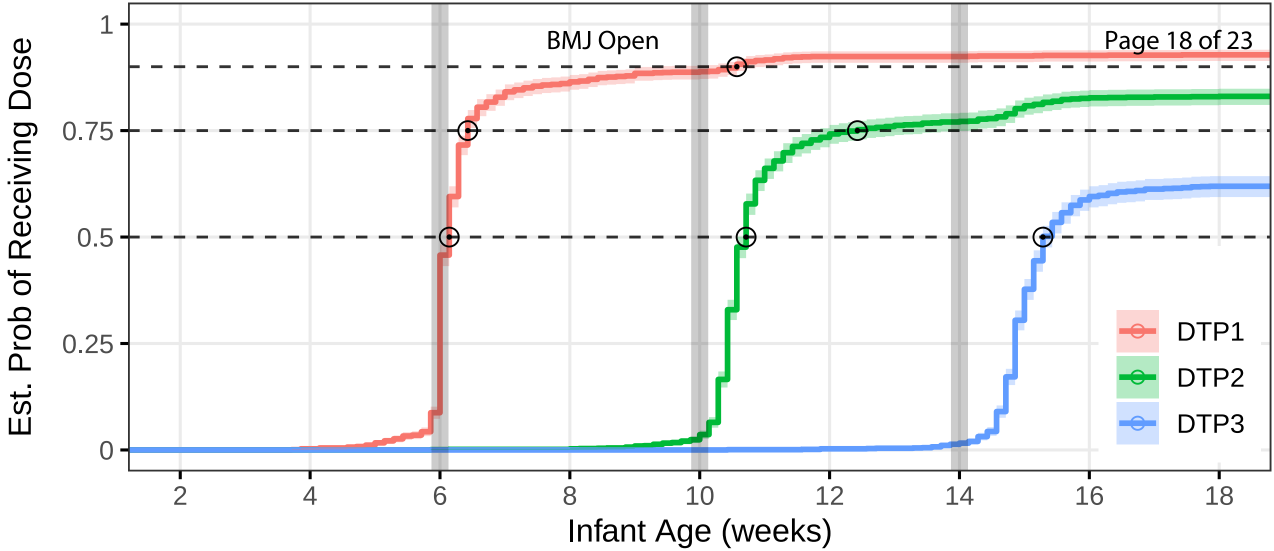

Attending & Eligible:

	2	4	6	8	10	12	14	16	18
DTP1	1497	1412	1276	118	77	12	7	3	1
DTP2	1497	1415	1399	1345	1275	182	111	10	2
DTP3	1497	1415	1400	1347	1310	1230	1086	62	3

Vaccinations (Cumulative):

	2	4	6	8	10	12	14	16	18
DTP1	0	4	685	1294	1330	1383	1383	1387	1389
DTP2	0	0	1	4	54	1111	1155	1238	1243
DTP3	0	0	0	0	0	4	24	891	927

Lost To Future Followup (Cumulative):

	2	4	6	8	10	12	14	16	18
DTP1	0	87	90	91	92	103	107	107	107
DTP2	0	88	115	159	189	218	236	251	253
DTP3	0	88	115	159	191	269	405	558	568

Infant Age (weeks)

For peer review only - http://bmjopen.bmj.com/site/about/guidelines.xhtml

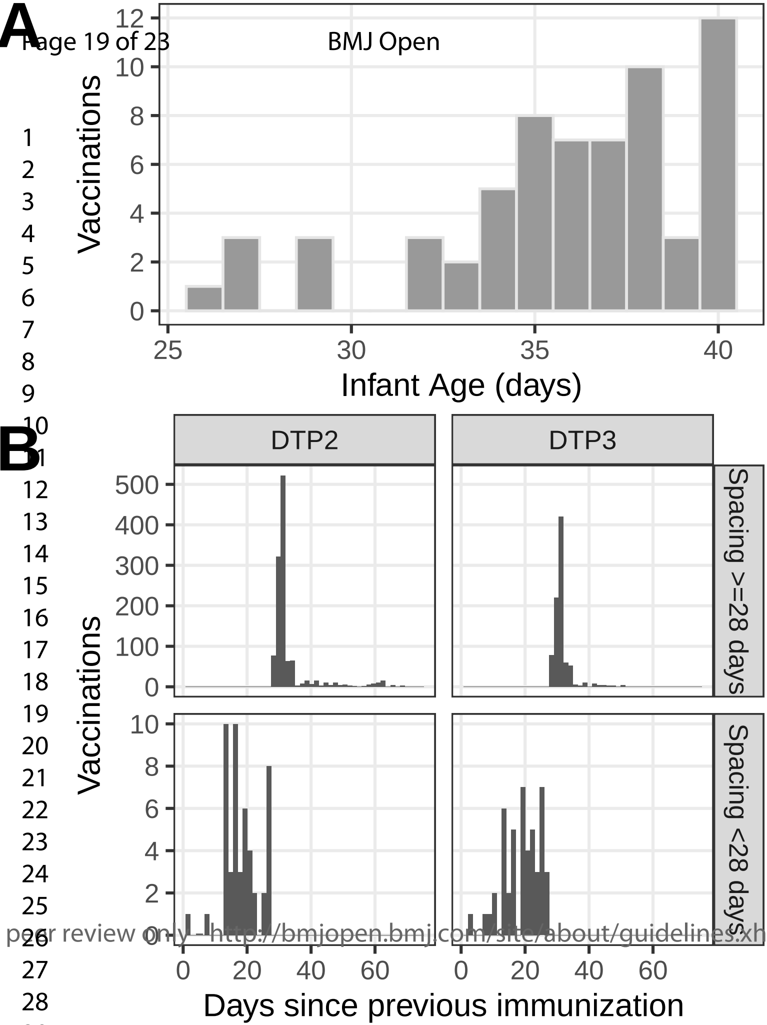

For peer review only - http://bmjopen.bmj.com/site/about/guidelines.xhtml

Implementation and adherence of routine pertussis vaccination (DTP) in a low-resource urban birth cohort

Gunning et al.

2020

Supplemental Figures and Tables

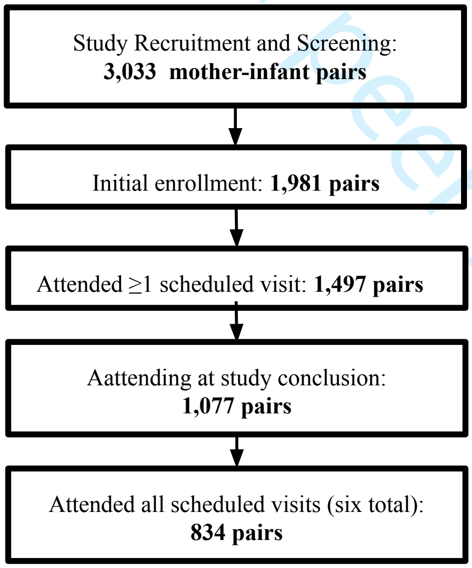

Figure S1: Study profile of birth cohort enrollment and attendance. Beyond eligibility and initial screening, the sole cause of cohort attrition was failure to attend one or more scheduled clinic visits.

For peer review only - http://bmjopen.bmj.com/site/about/guidelines.xhtml

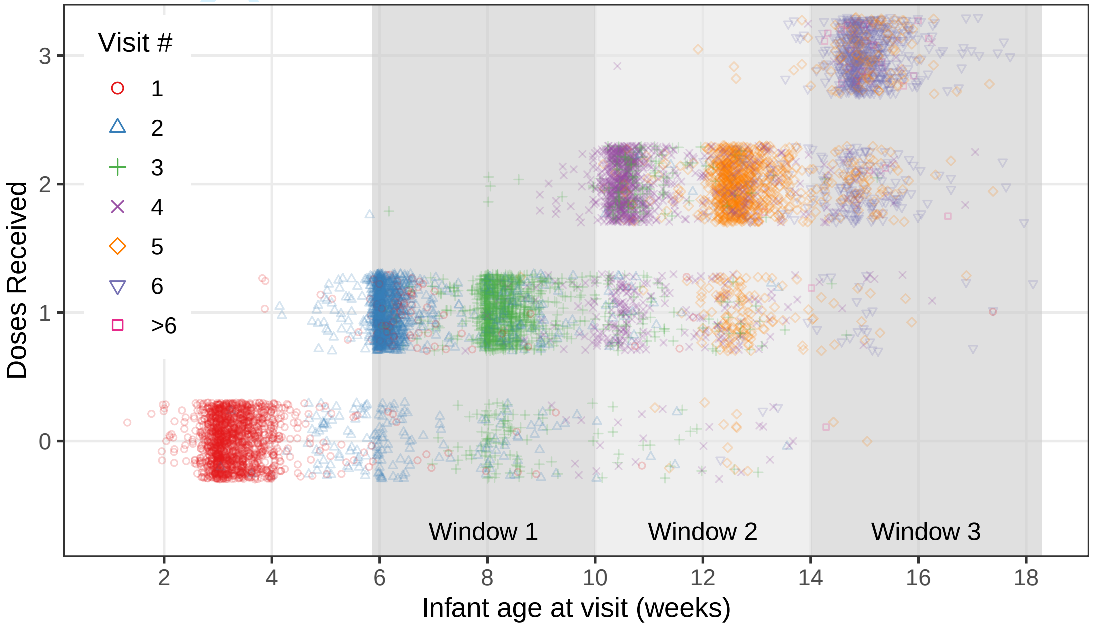

Figure S2: Infants were scheduled for 6 clinic visits over the course of the study. Figure shows the cumulative doses of DTP vaccine received per infant at each visit (color, shape) by infant age.

For peer review only - http://bmjopen.bmj.com/site/about/guidelines.xhtml

STROBE Statement—Checklist of items that should be included in reports of ***cohort studies***

	Item No	Recommendation
**Title and abstract**	1	(*a*) Indicate the study's design with a commonly used term in the title or the abstract **Title, "birth cohort", p1**
		(*b*) Provide in the abstract an informative and balanced summary of what was done and what was found **Abstract, sections Methods and Results, p1**
**Introduction**		
Background/rationale	2	Explain the scientific background and rationale for the investigation being reported **Intro, Paras 1-3, p2**
Objectives	3	State specific objectives, including any prespecified hypotheses **Intro, Paras 3 & 5, p 2-3**
**Methods**		
Study design	4	Present key elements of study design early in the paper **Methods, section Study Design, p 3-4**
Setting	5	Describe the setting, locations, and relevant dates, including periods of recruitment, exposure, follow-up, and data collection **Methods, section Study Design, p3**
Participants	6	(*a*) Give the eligibility criteria, and the sources and methods of selection of participants. Describe methods of follow-up **Methods, section Study Design, p3**
		(*b*) For matched studies, give matching criteria and number of exposed and unexposed **NA**
Variables	7	Clearly define all outcomes, exposures, predictors, potential confounders, and effect modifiers. Give diagnostic criteria, if applicable **Methods, section Assessment, p4**
Data sources/ measurement	8*	For each variable of interest, give sources of data and details of methods of assessment (measurement). Describe comparability of assessment methods if there is more than one group **Methods, section Assessment, p4**
Bias	9	Describe any efforts to address potential sources of bias **Methods, section Data and Statistical Analysis, last para, p5**
Study size	10	Explain how the study size was arrived at **Previously described (citation 18), p3**
Quantitative variables	11	Explain how quantitative variables were handled in the analyses. If applicable, describe which groupings were chosen and why **Methods, section Data and Statistical Analysis, p5**
Statistical methods	12	(*a*) Describe all statistical methods, including those used to control for confounding **Methods, section Data and Statistical Analysis, p5**
		(*b*) Describe any methods used to examine subgroups and interactions **Methods, section Data and Statistical Analysis, p5**
		(*c*) Explain how missing data were addressed **Methods, section Data and Statistical Analysis, p5**
		(*d*) If applicable, explain how loss to follow-up was addressed **Methods, section Data and Statistical Analysis, p5**
		(*e*) Describe any sensitivity analyses **NA**

For peer review only - http://bmjopen.bmj.com/site/about/guidelines.xhtml

**Results**		
Participants	13*	(a) Report numbers of individuals at each stage of study—eg numbers potentially eligible, examined for eligibility, confirmed eligible, included in the study, completing follow-up, and analysed<br>**Results, Section Participation, Fig 1A, Table 1, Figure S1, p5-6**
		(b) Give reasons for non-participation at each stage<br>**NA**
		(c) Consider use of a flow diagram<br>**Figure S1**
Descriptive data	14*	(a) Give characteristics of study participants (eg demographic, clinical, social) and information on exposures and potential confounders<br>**Results, Section Demographic Characteristics, Table 1, Figure 2, p6**
		(b) Indicate number of participants with missing data for each variable of interest<br>**Figure 1A, Figure 3**
		(c) Summarise follow-up time (eg, average and total amount)<br>**Results, section Participation, p4-5**
Outcome data	15*	Report numbers of outcome events or summary measures over time<br>**Figure 1 & 3, Tables 2-4, p7-8**
Main results	16	(*a*) Give unadjusted estimates and, if applicable, confounder-adjusted estimates and their precision (eg, 95% confidence interval). Make clear which confounders were adjusted for and why they were included<br>**Figure 1 & 3, Tables 2-4, p7-8**
		(*b*) Report category boundaries when continuous variables were categorized<br>**NA**
		(*c*) If relevant, consider translating estimates of relative risk into absolute risk for a meaningful time period<br>**NA**
Other analyses	17	Report other analyses done—eg analyses of subgroups and interactions, and sensitivity analyses<br>**Figure 4**
**Discussion**		
Key results	18	Summarise key results with reference to study objectives<br>**Discussion, paras 1-3, p 9**
Limitations	19	Discuss limitations of the study, taking into account sources of potential bias or imprecision. Discuss both direction and magnitude of any potential bias<br>**Discussion, paras 4-5, p10**
Interpretation	20	Give a cautious overall interpretation of results considering objectives, limitations, multiplicity of analyses, results from similar studies, and other relevant evidence<br>**Discussion, paras 7-8, p10-11**
Generalisability	21	Discuss the generalisability (external validity) of the study results<br>**Discussion, para 7&9, p10-11**
**Other information**		
Funding	22	Give the source of funding and the role of the funders for the present study and, if applicable, for the original study on which the present article is based<br>**Section Funding, p11**

*Give information separately for exposed and unexposed groups.

For peer review only - http://bmjopen.bmj.com/site/about/guidelines.xhtml

Note: An Explanation and Elaboration article discusses each checklist item and gives methodological background and published examples of transparent reporting. The STROBE checklist is best used in conjunction with this article (freely available on the Web sites of PLoS Medicine at http://www.plosmedicine.org/, Annals of Internal Medicine at http://www.annals.org/, and Epidemiology at http://www.epidem.com/). Information on the STROBE Initiative is available at http://www.strobe-statement.org.

BMJ Open

Implementation and adherence of routine pertussis vaccination (DTP) in a low-resource urban birth cohort

Journal:	*BMJ Open*
Manuscript ID	bmjopen-2020-041198.R2
Article Type:	Original research
Date Submitted by the Author:	13-Nov-2020
Complete List of Authors:	Gunning, Christian ; University of Georgia, Odum School of Ecology<br>Mwananyanda, Lawrence; Right to Care, Zambia<br>MacLeod, William; Boston University School of Public Health, Department of Global Health<br>Mwale, Magdalene; Right to Care, Zambia<br>Thea, Donald; Boston University School of Public Health, Department of Global Health<br>Pieciak, Rachel; Boston University School of Public Health, Department of Global Health<br>Rohani, Pejman; University of Georgia, Odum School of Ecology<br>Gill, Christopher; Boston University School of Public Health, Department of Global Health
**Primary Subject Heading**:	Infectious diseases
Secondary Subject Heading:	Public health, Paediatrics, Health informatics, Global health, Epidemiology
Keywords:	Public health < INFECTIOUS DISEASES, Community child health < PAEDIATRICS, Paediatric infectious disease & immunisation < PAEDIATRICS, Epidemiology < INFECTIOUS DISEASES

SCHOLARONE™
Manuscripts

I, the Submitting Author has the right to grant and does grant on behalf of all authors of the Work (as defined in the below author licence), an exclusive licence and/or a non-exclusive licence for contributions from authors who are: i) UK Crown employees; ii) where BMJ has agreed a CC-BY licence shall apply, and/or iii) in accordance with the terms applicable for US Federal Government officers or employees acting as part of their official duties; on a worldwide, perpetual, irrevocable, royalty-free basis to BMJ Publishing Group Ltd ("BMJ") its licensees and where the relevant Journal is co-owned by BMJ to the co-owners of the Journal, to publish the Work in this journal and any other BMJ products and to exploit all rights, as set out in our licence.

The Submitting Author accepts and understands that any supply made under these terms is made by BMJ to the Submitting Author unless you are acting as an employee on behalf of your employer or a postgraduate student of an affiliated institution which is paying any applicable article publishing charge ("APC") for Open Access articles. Where the Submitting Author wishes to make the Work available on an Open Access basis (and intends to pay the relevant APC), the terms of reuse of such Open Access shall be governed by a Creative Commons licence – details of these licences and which Creative Commons licence will apply to this Work are set out in our licence referred to above.

Other than as permitted in any relevant BMJ Author's Self Archiving Policies, I confirm this Work has not been accepted for publication elsewhere, is not being considered for publication elsewhere and does not duplicate material already published. I confirm all authors consent to publication of this Work and authorise the granting of this licence.

For peer review only

Implementation and adherence of routine pertussis vaccination (DTP) in a low-resource urban birth cohort

April 1, 2020

Authors: CE Gunning*[1], L Mwananyanda[2], WB Macleod[3], M Mwale[2], DM Thea[3], RC Pieciak[3], P Rohani[1], and CJ Gill[3]

* Corresponding author (email: research@x14n.org)

[1] University of Georgia, Odum School of Ecology

[2] Right To Care Zambia

[3] Boston University School of Public Health, Department of Global Health

For peer review only

Abstract

Introduction

Reliable information on rates of up-to-date coverage and timely administration of routine childhood immunizations is critical for guiding public health efforts worldwide, yet prospective observation of vaccination programs within individual communities is rare. Here we provide a longitudinal analysis of the directly-observed administration of a 3-dose primary vaccination series to infants in a low-resource community in Lusaka, Zambia.

Methods

Throughout 2015, we recruited a longitudinal birth cohort of mother/infant pairs (initial enrollment, 1,981 pairs; attending, 1,497 pairs) from the peri-urban informal settlement of Chawama compound, located in Lusaka, Zambia. We prospectively monitored the administration of scheduled Diphtheria-Tetanus-Pertussis (DTP) vaccinations across the first 14-18 weeks of life. We analyzed study attendance and vaccine coverage, both overall and stratified by age group. We employed Kaplan-Meier analyses to estimate delays in age-appropriate administration of vaccine doses. We also assessed schedule timing violations, including early and compressed dose administration.

Results

At study completion, first dose (DTP1) rates were high (92.9% of attending), whereas third dose completion (DTP3) rates were far lower (61.9%). Missed vaccinations and study dropout both contributed to the low DTP3 completion rates. DTP1 was administered very late (at or after 10 weeks) to 61 infants (4.1%). DTP1 was administered too early to 64 infants (4.3%), and 77 (5.1%) received consecutive doses below the minimum recommended spacing of 28 days.

Conclusions

We observe substantial individual variation in the timing of early childhood DTP doses, though following this birth cohort proved challenging. Our results indicate that timely administration of both DTP1 and DTP3 remains a challenge in this community. These directly-observed, individual-based results provide an important counterpoint to more course-grained, survey-based national and province estimates of up-to-date vaccine coverage. This study also highlights the challenges of vaccine hesitancy and sub-optimal utilization of (no-cost) healthcare services in a low-resource urban setting.

Strengths and limitations of this study

- To our knowledge, this is the first prospective urban birth cohort study in a low-resource setting to directly document vaccinations at the place and time of administration.
- By following individuals across time, we are better able to assess healthcare utilization behavior relative to cross-sectional surveys.
- This study provides a detailed view of a single community across a year, and may not capture long-term trends or generalize to other populations.

Introduction

Estimates of vaccine coverage play a central role in epidemiology and public health, from identifying at-risk communities and age groups for supplementary immunization activities (SIAs) [1, 2] to their use in disease transmission models and subsequent inference [3–5]. Coverage estimates are also key indicators of national vaccination implementation success [6]. As such, high-quality vaccine coverage estimates are critical in shaping modern public health policy.

Estimates of childhood *up-to-date* vaccine coverage from cross-sectional studies (e.g., Demographic and Health Surveys (DHS) [7] and UNICEF [8]) have revealed numerous factors affecting vaccine administration in low- and middle-income countries (LMICs) [9–13]. However, these coarse-grained estimates can mask complexities in compliance and implementation. Particularly in LMICs, spatial and socioeconomic heterogeneities in coverage are common, as are delays in the timely administration of vaccine doses [9, 14]. Further, the reliability of surveys based on personal and informal records, i.e., vaccine cards and maternal recall, remains unclear [15].

With few exceptions [16, 17], detailed observations of childhood vaccinations during routine clinical care are lacking. Granular, community-based observations of individual patient histories can reveal temporal patterns in schedule adherence, including delays in age-appropriate vaccinations. Prospective observation of a cohort can also reveal if community-level DTP coverage differs substantially from regional coverage, and can help identify behavioral factors that affect the success of vaccine implementation programs, including vaccine hesitancy.

For peer review only - http://bmjopen.bmj.com/site/about/guidelines.xhtml

Here we provide a detailed analysis of infant vaccination in a sub-Saharan low-resource urban setting in Lusaka, Zambia. Throughout 2015, we followed a longitudinal birth cohort of mother-infant pairs from birth through 14 weeks of age as part of a broader study of pertussis transmission. Participants were scheduled for 6 clinic visits total; the primary 3-dose DTP series was administered at three of these visits according to the Zambian national schedule of 6, 10, and 14 weeks of age. Our team prospectively documented all clinic visits and administration of DTP doses using an electronic data capture system [18]. We quantified the DTP series dose trajectory of individual infants, and assessed the proportion of infants with up-to-date vaccinations, or who missed one or more doses. We evaluated compliance with published vaccine schedule guidelines, including minimum age restrictions and dose spacings.

In short, our use of directly-observed age-specific DTP administration data in a typical LMIC community clinic offers a unique window into vaccine implementation at the individual patient level that can help inform vaccine policy decisions globally.

Methods

Study design

The Southern Africa Mother Infant Pertussis Study (SAMIPS) was a longitudinal birth cohort study conducted in Lusaka, Zambia from March 2015-February 2016 to assess the incidence of pertussis in a representative population of otherwise healthy infants (details provided elsewhere [18]). Mother–infant pairs were enrolled from the population of the Chawama compound, a densely populated peri-urban slum of approximately 150,000 residents near central Lusaka. The study goal was to capture all live births that occurred in Chawama compound during the study period and, prior to study initiation, a public outreach campaign provided pregnant Chawama residents with information about the study. Infants were enrolled at the Chawama Primary Health Clinic (PHC) during their first scheduled postpartum well-child visit (at approximately 1 week old). The PHC is the only government-supported clinic in this community and, as such, is the primary source of medical care for Chawama residents. This feature allowed us to maximize study reach by capturing the majority of participant health care consumption in this community during the study period from a single point of service.

Enrollment eligibility required that infants were born after 37 weeks, weighed over 2500 grams, and were delivered without complications or apparent disease. Eligibility also required signed consent, Chawama residency (anticipated remaining in the community during study period), that the HIV status of mothers was known prior to delivery, and that HIV+ mothers were receiving prophylactic antiretroviral therapy at the time of delivery [18]. An extended discussion of study size is provided in [18].

Mothers were incentivized to join and remain in the cohort in three ways. First, the SAMIPS medical staff provided all routine and acute medical care for study participants during their time of enrollment. This significantly reduced clinic waiting times for care from over 3 hours to half an hour or less. Second, mothers received a travel stipend for each visit

For peer review only - http://bmjopen.bmj.com/site/about/guidelines.xhtml

valued at approximately 7 US dollars. Lastly, a small gift of baby supplies was provided for mothers attending the final scheduled study visit.

Enrolled infants were scheduled for six routine clinic visits at 2-3 week intervals through 14 weeks old (maximum 18 weeks). At each clinic visit, nasopharyngeal (NP) swabs were obtained from both mother and infant, and possible pertussis symptoms were recorded. Regular clinic staff provided routine childhood vaccinations at no cost. Administration of DTP doses 1-3 was scheduled at visits corresponding to 6, 10, and 14 weeks of age according to Zambian national guidelines (see WHO Expanded Programme of Immunization (EPI) schedule [19]). DTP vaccinations were administered as a pentavalent combination (Pentavac, Serum Institute of India Limited, Pune, India) that included whole-cell pertussis, *Haemophilus influenzae* type B (HIB), and Hepatitis B. The pneumococcal conjugate vaccine (PCV10) was co-administered with DTP; oral rotavirus vaccine administration was scheduled for 6 and 10 weeks of age.

At each visit, infant vaccination cards ("under 5 cards") were reviewed by clinic staff, and the dates of any previously unrecorded vaccinations were noted. When participants missed a scheduled visit, clinic staff attempted to contact participants by phone to reschedule the visit.

Patient and Public Involvement

This research was done without patient involvement. Patients were not invited to comment on the study design and were not consulted to develop patient relevant outcomes or interpret the results. Patients were not invited to contribute to the writing or editing of this document for readability or accuracy.

Assessment of Vaccine Administration

For protection against pertussis, the WHO recommends that infants receive a 3-dose primary series with a DTP-containing vaccine [19]. Guidelines specify a minimum administration age of 6 weeks for the first dose (DTP1), a minimum interval of 4 weeks between subsequent doses, and a target inter-dose interval of 4-8 weeks. In Zambia, pregnant mothers receive one or more doses of tetanus toxoid monovalent vaccine during pregnancy.

For this analysis, we use the Zambian 6-10-14 week EPI schedule for DTP to define target age windows in which infants should have received each DTP dose: window 1, 41-69 days; window 2, 70-97 days; window 3, 98-126 days. Note that window 1 extends one day early to account for uncertainty the exact timing birth and vaccination. We tabulate the number of doses each infant received in each window, and at study completion. For example, an infant might receive no doses during window 1, and a dose in both windows 2 and 3, for a total of two doses at study completion.

Infants who received all scheduled doses to-date were categorized as *up-to-date*. However, once a window was missed, an infant was no longer up-to-date during subsequent age windows. Infants who received no doses by the end of the dose window were categorized

For peer review only - http://bmjopen.bmj.com/site/about/guidelines.xhtml

as *unvaccinated*. Vaccinations that occurred >1 day prior to window 1 were classified as *early*. Vaccinations administered less than 4 weeks after a preceding dose were classified as *compressed*.

Data and Statistical Analyses

Administration of all DTP doses by clinic staff during the study period was directly observed and documented by study staff. All data were manually recorded on standardized data sheets, and digitized using a digital pen electronic data capture system (XCallibre, Durban, South Africa. https://www.xcallibre.com). Data were de-identified prior to analysis: each mother-infant pair was assigned a random date offset of +/-3 days, and all study dates (i.e., birth and visits) were adjusted by this offset.

Mother-infant pairs were considered *attending* (on-study) from enrollment until their last recorded clinic visit during the study period. We exclude from further consideration individuals who enrolled in SAMIPS but did not attend any scheduled clinic visits.

We report the number of infants who received exactly (and at least) 0-3 doses by study completion, along with the percent of attending study infants in each of these groups.

We stratified dose outcomes by age window. For each window, we report the number of *unique* infants attending at least one scheduled visit. We also report the number of infants who: received or missed a vaccine dose, received compressed or early vaccinations, and were up-to-date (along with respective percentages). Since attrition and irregular attendance are expected in cohort studies, we compute percentages relative to the number of unique infants in each window (above).

We perform a modified Kaplan-Meier analysis of vaccination delays [20, 14]. The standard Kaplan-Meier method assumes that non-attending individuals (i.e., right-censored) experience events at the same rate as attending individuals. Here we explore an alternative worst-case scenario where non-attending individuals receive no further vaccinations during the study period (subsequent to study drop-out). While subsequent make-up vaccinations may occur, our assumption of minimal off-study vaccination is plausible, as the Chawama clinic is the sole provider of DTP in this community, and study staff recorded all DTP doses there during the study period.

Results

Participation

Infants were enrolled in the study between March and December 2015 (1,981 out of 3,033 screened). The five most common reasons for study exclusion include lack of consent (14.3%), low infant birth weight (7.9%), community non-residence (5.9%), mother anticipates moving (4.1%), and mother not 18-39 years old (3.7%) (see Supplemental Table 3 in [18] for enrollment details). Enrollees that did not attend any subsequent clinic visits (484 total) were removed from this analysis, leaving 1,497 initially attending infants. We depict study participation over time of the remaining infants in Figure 1A, while Figures 1B-

For peer review only - http://bmjopen.bmj.com/site/about/guidelines.xhtml

C detail the timing of study events. At the beginning of windows 1-3, 1,400 infants (window 1), 1,306 infants (window 2), and 1,089 infants (window 3) were still attending (see Figure S1 for study profile). The observed drop-off in cohort size near the end of window 3 represents graduation from the study, as infants' final clinic visits (and DTP dose) were scheduled for approximately 14 weeks of age. Overall, the duration of study attendance was a median of 96 days (min=12 days, max=120 days, excluding one recorded vaccination at age 272 days).

Demographic Characteristics

Overall, the demographic characteristics of participants did not co-vary with duration of study attendance (see Table 1), including all enrolled individuals, individuals attending at least one scheduled visits (attending), and individuals attending all scheduled visits (completed). Mothers' median age was 25 years, regardless of attendance. Across enrolled mothers, 17.5% were HIV+, while slightly more attending mothers were HIV+ (19.3%). Infants' median birth weight was 3000 grams. Regardless of attendance, median household size was 1 infant of <1 year of age (including study infant) and 2 children of <5 years of age (including infants).

With one exception, demographic characteristics did not affect the relative risk of the following end-of-study outcomes: being unvaccinated, receiving at least one dose (DTP1), and receiving all three doses (DTP3) (Figure 2). We did observe a marginally lower risk of DTP3 (i.e., higher risk of incomplete vaccination) in infants who received OPV at birth (RR=0.89, 95% CI: 0.81-0.97). However, we observed no consistent direction of associations, and we note that the observed associations are consistent with sampling variation arising in multiple comparisons.

Table 1: **Demographic characteristics of participants** by duration of study attendance (columns), showing percentage or median, as appropriate. Interquartile range is shown in parentheses. **Attending** mother-infant pairs attended one or more scheduled clinic visit. **Completed** individuals attended all six scheduled visits.

	Duration of		
Parameter	Enrolled	Attending	Completed
Number Under Study	1981	1497	834
**Mothers**			
Married	90.2%	90.1%	90.3%
HIV+	17.5%	19.3%	18.6%
Median Age	25 (21, 29)	25 (22, 29)	25 (22, 30)
Median Infants In House (<1 year)	1 (1, 1)	1 (1, 1)	1 (1, 1)
Median Children In House (< 5 years)	2 (1, 2)	2 (1, 2)	2 (1, 2)
**Infants**			
Born at Chawama PHC	56.9%	56.7%	58.2%
Born at University Teaching Hospital	34.8%	35.5%	34.3%
Female Sex	46.9%	46.4%	46.4%

For peer review only - http://bmjopen.bmj.com/site/about/guidelines.xhtml

Received BCG at birth	46.4%	46.6%	46.2%
Received OPV at birth	33.4%	32.4%	27.7%
Median Birth Weight (kg)	3 (2.8, 3.3)	3 (2.8, 3.3)	3.1 (2.8, 3.3)

Scheduled Doses

Vaccination outcomes at the completion of the study (total doses received) are summarized in Table 2, while Table 3 details the cumulative doses received at the end of each age window. Overall, approximately 7.1% (107/1,497) of attending infants had not received any DTP vaccinations at the conclusion of the study (Table 2). This corresponds to 9.8% (107/1,089) of infants who remained in the cohort up to the beginning of window 3 (and thus had the opportunity to receive each of the three scheduled doses, see Figure 1A).

The timing of DTP dose administration to individual participants is shown in Figure 1B (for a detailed view of all clinic visits, see Figure S2). We also aggregate these data by week, highlighting that most infants received each DTP dose within a week of the target schedule of 6, 10, and 14 weeks of age (Figure 1C). However, deviations from the planned schedule were common. For example, early DTP vaccinations were administered to 64 infants prior to window 1 (Table 3), before which no vaccinations should have occurred (except for doses of the oral polio and BCG vaccines administered at birth, see Table 1). Similarly, the spread of events to the right of the target administration age of 6, 10 and 14 weeks represents vaccination after the scheduled delivery date (Fig 1B-1C). Two general trends here are apparent across the three scheduled doses. With each subsequent dose: 1), the average time of administration drifts farther to the right of (later than) the target schedule; and 2) the clustering of administration times becomes more dispersed.

We also observed infants who missed a dose and later received a subsequent dose on (or near) schedule (Figures 1B-C). For example, 118 infants received their first DTP dose during window 2 , while 170 infants received a second DTP dose during window 3 (Table 3, Figure 1C). Since the study did not follow participants beyond age 16 weeks (with few exceptions), it remains unclear whether (and when) catch-up of any doses missed during the study might have occurred.

Time-stratified results based on infant visits within each age window are shown in Table 4. These results are less sensitive to cohort attrition, as they assess outcomes over a shorter time period. Here, we find that up-to-date rates fell across the study from 95.8% of visiting infants (window 1, DTP1) to 88.4% (window 2, DTP2) and finally to 83.7% (window 3, DTP3). The unvaccinated rate of visiting infants also fell from window 1 (12.3%) to window 2 (8.8%), but did not decrease further during window 3 (9.9%). We note that many infants who visited the clinic in windows 1-3 nonetheless missed scheduled vaccinations:6.6% (window 1), 9.1% (window 2), and 8.4% window 3.

Table 2: **Vaccine status at study completion.** Number of infants that received at least (or exactly) 0-3 doses of DTP at study completion. Percentages are rounded (out of 1,497 attending infants).

For peer review only - http://bmjopen.bmj.com/site/about/guidelines.xhtml

N	Exactly N Doses (%)	At Least N Doses (%)
**0**	107 (7.1)	1497 (100.0)
**1**	147 (9.8)	1390 (92.9)
**2**	316 (21.1)	1243 (83.0)
**3**	927 (61.9)	927 (61.9)

Table 3: **Cumulative doses received across study.** Number of unique infants that received DTP1-3 by the *end* of each age window (columns, in days). Bottom row shows total doses administered in each window (some infants received more than one dose per window). A single infant received DTP1 at age 272 days (not shown).

	**Early**	**Window 1**	**Window 2**	**Window 3**
**Received**	**0-40**	**41-69**	**70-97**	**98-127**
**DTP1**	64	1264	55	6
**DTP2**		36	1117	90
**DTP3**			20	907
**Any**	64	1300	1192	1003

Table 4: **Vaccinations stratified by age window,** showing number of unique infants *within* each age window. **Visits:** infants attending ≥1 scheduled visit. **Received, Missed:** infants that received (or did not receive) a DTP dose. **Compressed:** infants vaccinated <4 weeks after previous dose (see also Fig. 4B). **Up-to-date:** infants that received the targeted N^{th} dose in this window. **Unvaccinated:** infants that received zero total doses by end of window. Percentages are rounded (out of **Visits**).

Age Window	Target (N)	Visits	Received (%)	Missed (%)	Compressed (%)	Up-to-date (%)	Unvaccinated (%)
**41-69**	**1**	1378	1287 (93.4)	91 (6.6)	-	1320 (95.8)	169 (12.3)
**70-97**	**2**	1301	1183 (90.9)	118 (9.1)	50 (3.8)	1150 (88.4)	114 (8.8)
**98-127**	**3**	1093	1001 (91.6)	92 (8.4)	47 (4.3)	915 (83.7)	108 (9.9)

For peer review only

Vaccine timing

A timely initial dose plays an important role in protecting infants from preventable morbidity and mortality [21]; prior to DTP1, study infants' protection against pertussis depended on unknown but presumably low and waning maternally-derived immunity. We observed 61 infants (4.1% of attending) that received DTP1 at 10 weeks of age or greater, though only 6 (0.4% of attending) received DTP1 at 14 weeks of age or greater (Table 3, Figure 3).

Our modified worst-caseKaplan-Meier survival analysis (assuming no vaccination in infants lost to follow-up) revealed a modest median delay in the timely administration of DTP1 (1 day), though the median delay grew across primary series to 5 days (DTP2) and 9 days (DTP3). However, many infants experienced more substantial delays: for DTP1 the upper decile delay (slowest 10%) was 32 days, and for DTP2 the upper quantile delay (slowest 25%) was 17 days. Finally, 570 infants (38.1% of attending) did not receive DTP3 during the study.

By comparison, schedule violations involving early and compressed doses were minor. As noted above, DTP1 was administered too early to 64 infants (4.3% of attending), with most of these doses administered 2-7 days early (at age 35-40 days) (Table 3, Figure 4A). The median spacing between subsequent doses (31 days) slightly exceeded the minimum recommended spacing of 28 days (Figure 4B). A small minority of infants experienced dose compression between doses 1-2 and/or doses 2-3: 77 infants received doses spaced less than 25 days apart, while 48 infants received doses less than 20 days apart.

Discussion

This study offers a descriptive analysis of prospectively-collected, carefully documented observations of DTP vaccinations administered in an urban African public health clinic. Overall, we found that 61.9% of attending infants (927/1,497) received the full DTP3 series in a timely manner, corresponding to 85.1% of infants who attended into window 3 (and were thus available for a third dose, 791/1,089). . This is remarkable given that Chawama's EPI implementation lacks direct client outreach or other supportive interventions. However, we were concerned by higher than anticipated rate of missed (and delayed) doses: at study completion, 9.8% of attending infants had received only one dose, while 7.1% of attending infants lacked records for any DTP vaccinations. In short, our findings suggest that access to (no-cost) healthcare, available to all study infants, was not the sole limiting factor in DTP uptake in Chawama compound.

While not directly comparable with our results, WHO-UNICEF estimated that Zambia's 2015 up-to-date DTP3 coverage was 90% [22], much higher than our findings of 61.9%. However, our DTP1 rates at study completion (92.9%) were proportionally closer to corresponding WHO-UNICEF estimates (97%) and 2013-2014 Zambia DHS estimates for Lusaka province (97.8%) [23] (though the ceiling effect leaves little range for observable differences here). Overall, our results suggest that delays in timely vaccinationwarrants further attention in Zambia, as does spatial heterogeneity (e.g., across socioeconomic gradients).

For peer review only - http://bmjopen.bmj.com/site/about/guidelines.xhtml

The longitudinal nature of this study allowed us to resolve key implementation features that cross-sectional or survey-based approaches cannot resolve, including dose timing. In this cohort, early and compressed vaccinations were relatively rare. However, we observed that administration of scheduled vaccinations was more variable and, on average, less timely with each subsequent dose. This pattern indicates that delays are not random across life, and suggests that delays early in life have down-stream effects on the timing of subsequent vaccinations. We also gained insights into how individuals' behavior affected population-level coverage rates. For example, the small change in unvaccinated infants attending window 2 (114, 8.8%) and window 3 (108, 9.9%) suggests that doses are not skipped randomly by study participants. Rather, a core group of individuals appears to have regularly attend clinic visits while opting out of (no-cost) routine vaccines, despite encouragement by our study team. Indeed, earlier qualitative work in this cohort suggests that vaccine hesitancy plays a non-trivial role here[24].

Following a birth cohort across almost five months of life proved challenging in this setting. We experienced high rates of initial non-participation among eligible infants (484 out of 1,981 initial enrollees), as well as ongoing attrition of the cohort throughout the study. Indeed, only 72.7% of attending infants (1,089, Figure 1) participated in the study up to an age of 14 weeks (98 days), and were thus available to receive all scheduled DTP doses during the study. However, our intensive prospective monitoring of the primary source of health care in Chawama leads us to believe that enrollees who missed one or more visits rarely receive unrecorded vaccinations at the PHC during the study period. In addition, we expect that non-clinic vaccinations of enrollees during this period were rare due to cost and geographical access. On the other hand, study involvement could have introduced an upwards bias on clinic visit behavior and DTP uptake relative to this population's background, non-study health care behavior.

Two unresolved questions remain: 1) whether infants who left the cohort early were subsequently vaccinated at the PHC after study graduation or were vaccinated elsewhere off-study, and 2) whether attending infants who missed scheduled doses later received catch-up doses, e.g., at subsequent well-child visits. In Lusaka, catch-up of missed doses is available at monthly clinic visits and two targeted "child health weeks" (June and November). However, these supplemental vaccination campaigns are implemented without respect to known vaccination compliance (as documented in each child's under five card), and thus do not systematically target children who missed doses during the primary series. Moreover, after the final study visit (at age 14-16 weeks), the next routine well child visit is not until nine months of age, when the measles vaccine dose is given. As such, even when supplemental vaccination occurs, the subsequent delays in timely vaccination may be substantial.

We believe this study fills an important gap in the childhood vaccination literature. As a periurban informal settlement, Chawama represents a typical under-served community in a LMIC, where a single public clinic is the primary health care resource for more than 150,000 residents. This study provides an important point of comparison for additional such prospective studies, in order to better assess variation by geographic region or resource

For peer review only - http://bmjopen.bmj.com/site/about/guidelines.xhtml

availability. We also believe that further investigation of these findings could provide significant value to the public health community. For example, while beyond the scope of this study, a cross-sectional seroconversion survey of young children (e.g., 1-5 years of age) could help quantify the frequency of late or absent vaccination stratified by age. For example, seropositivity for tetanus antibodies could provide a robust indicator of (at least) DTP1 uptake, as tetanus toxoid is highly immunogenic and elicits durable immunity [25]. Such surveys could help identify at-risk communities and target additional resources for catch-up campaigns.

Despite lingering uncertainties, our analyses raise broad questions about pertussis control in low-resource settings, including herd immunity and epidemic preparedness. Crowded living situations can facilitate pertussis transmission relative to less dense regions [26, 27], while the scarcity of macrolide treatments in this community may yield longer-lasting infections [28]; both effects could raise the critical vaccination threshold required for herd immunity [29]. Further, lower DTP coverage here relative to Lusaka province is consistent with the previously-described impacts of socioeconomic status [10–13]. Together, these forces could allow dense informal settlements to serve as foci for more widespread pertussis epidemics. Our results also highlight the potential public health consequences of such an epidemic. We directly observed 169 infants (11.3% of attending) who remained unvaccinated at age 10 weeks, along with a corresponding 114 infants (7.6%) at age 14 weeks. These infants were at elevated risk of severe disease, hospitalization, and death [30, 31], yet low-resource settings such as Chawama are ill-prepared to treat severe cases or high case loads. Indeed, the PHC has only 10 hospital beds to serve this community.

These results have additional implications for the full package of vaccines administered via the EPI schedule, as failure to receive a scheduled DTP dose may also indicate failure to receive additionally scheduled vaccinations. For example, both the pneumococcal conjugate and rotavirus vaccines are commonly co-administered with DTP. We expect that marginal improvements in EPI implementation would be highly impactful to public health outcomes, particularly in under-served communities, and that detailed descriptions of when and why EPI schedule violations occur will help identify and remediate existing barriers to vaccine uptake in these communities.

In summary, this study provides a uniquely granular view of routine DTP implementation in a low-resource urban African setting that highlights individual behavior. Our data suggest that delayed age of first vaccination, along with possible vaccine hesitancy and loss of herd immunity, poses a significant public health risk to this community, echoing calls to strengthen EPI programs in low-resource urban settings [32, 33].

Competing Interests

The authors declare no competing interests.

For peer review only - http://bmjopen.bmj.com/site/about/guidelines.xhtml

Data Sharing

Extra data can be accessed via the Dryad data repository at http://datadryad.org/ with the doi: 10.5061/dryad.mpg4f4qxs

Author Contributions

Conceived study: CJG. Designed study: CJG, CEG. Conducted analysis, created figures and tables: CEG. Contributed to and interpreted analysis: CEG, WBM, PR, CJG. Wrote manuscript: CEG, PR, CJG. Contributed to manuscript revisions: CEG, LM, WBM, MM, DMT, RCP, PR, CJG.

Funding

The Bill & Melinda Gates Foundation funded the initial SAMIPS cohort (OPP 1105094). The National Institutes of Health/National Institute of Allergy and Infectious Diseases supported all subsequent analyses, including this analysis, using the SAMIPS database (1R01 AI133080). The study sponsors had no role in study design, analysis, or publication.

References

[1] Marita Zimmermann, Kurt Frey, Brittany Hagedorn, AJ Oteri, Abdulazeez Yahya, Maimuna Hamisu, Fred Mogekwu, Faisal Shuaib, Kevin A McCarthy, and Guillaume Chabot-Couture. Optimization of frequency and targeting of measles supplemental immunization activities in Nigeria: A cost-effectiveness analysis. *Vaccine*, 37 (41):6039–6047, 2019.

[2] Maria Joyce U Ducusin, Maricel de Quiroz-Castro, Sigrun Roesel, Luzviminda C Garcia, Dulce Cecilio-Elfa, W William Schluter, James L Goodson, and Eugene Lam. Using the World Health Organization measles programmatic risk assessment tool for monitoring of supplemental immunization activities in the Philippines. *Risk Analysis*, 37(6):1082–1095, 2017.

[3] Manoj Gambhir, Thomas A Clark, Simon Cauchemez, Sara Y Tartof, David L Swerdlow, and Neil M Ferguson. A change in vaccine efficacy and duration of protection explains recent rises in pertussis incidence in the United States. *PLoS Comput Biol*, 11(4):e1004138, 2015.

[4] Yoon Hong Choi, Helen Campbell, Gayatri Amirthalingam, Albert Jan Van Hoek, and Elizabeth Miller. Investigating the pertussis resurgence in England and Wales, and options for future control. *BMC medicine*, 14(121): 1–11, 2016.

[5] Matthieu Domenech de Cell`es, Felicia M. G. Magpantay, Aaron A. King, and Pejman Rohani. The impact of past vaccination coverage and immunity on pertussis resurgence. *Science Translational Medicine*, 10(434), 2019. ISSN 1946-6234. doi: 10.1126/scitranslmed.aaj1748. URL https://stm.sciencemag.org/content/10/434/eaaj1748.

[6] Jean-Marie Okwo-Bele and Thomas Cherian. The Expanded Programme on Immunization: a lasting legacy of smallpox eradication. *Vaccine*, 29:D74–D79, 2011.

[7] Madeleine Short Fabic, YoonJoung Choi, and Sandra Bird. A systematic review of demographic and health surveys: data availability and utilization for research. *Bulletin of the World Health Organization*, 90:604–612, 2012.

[8] Anthony Burton, Roeland Monasch, Barbara Lautenbach, Marta Gacic-Dobo, Maryanne Neill, Rouslan Karimov, Lara Wolfson, Gareth Jones, and Maureen Birmingham. WHO and UNICEF estimates of national infant immunization coverage: methods and processes. *Bulletin of the World Health Organization*, 87:535–541, 2009. URL https://www.who.int/bulletin/volumes/87/7/08-053819/en/.

[9] Andrew Clark and Colin Sanderson. Timing of children's vaccinations in 45 low-income and middle-income countries: an analysis of survey data. *The Lancet*, 373(9674):1543–1549, 2009.

[10] Mary-Christine Sullivan, Ayalew Tegegn, Fasil Tessema, Sandro Galea, and Craig Hadley. Minding the immunization gap: family characteristics associated with completion rates in rural Ethiopia. *Journal of Community Health*, 35(1):53–59, 2010.

[11] Ahmad Reza Hosseinpoor, Nicole Bergen, Anne Schlotheuber, Marta Gacic-Dobo, Peter M Hansen, Kamel Senouci, Ties Boerma, and Aluisio JD Barros. State of inequality in Diphtheria-Tetanus-Pertussis immunisation coverage in low-income and middle-income countries: a multi-country study of household health surveys. *The Lancet Global Health*, 4(9): e617–e626, 2016.

For peer review only

[12] Jin-Won Noh, Young-mi Kim, Nabeel Akram, Ki-Bong Yoo, Jumin Park, Jooyoung Cheon, Young Dae Kwon, and Jelle Stekelenburg. Factors affecting complete and timely childhood immunization coverage in Sindh, Pakistan; a secondary analysis of cross-sectional survey data. *PloS one*, 13(10):e0206766, 2018.

[13] Ikuma Nozaki, Masahiko Hachiya, and Tomomi Kitamura. Factors influencing basic vaccination coverage in Myanmar: secondary analysis of 2015 Myanmar demographic and health survey data. *BMC public health*, 19(1): 242, 2019.

[14] Manas K Akmatov and Rafael T Mikolajczyk. Timeliness of childhood vaccinations in 31 low and middle-income countries. *J Epidemiol Community Health*, 66(7):e14–e14, 2012.

[15] Melody Miles, Tove K Ryman, Vance Dietz, Elizabeth Zell, and Elizabeth T Luman. Validity of vaccination cards and parental recall to estimate vaccination coverage: a systematic review of the literature. *Vaccine*, 31 (12):1560–1568, 2013.

[16] Shankar Prinja, Madhu Gupta, Amarjeet Singh, and Rajesh Kumar. Effectiveness of planning and management interventions for improving age-appropriate immunization in rural India. *Bulletin of the World Health Organization*, 88:97–103, 2010. doi: 10.2471/BLT.08.059543.

[17] Ayebo Evawere Sadoh, Damian Uchechukwu Nwaneri, Bamidele Charity Ogboghodo, and Wilson Ehidiamen Sadoh. Effect of introduction of pentavalent vaccine as replacement for Diphtheria–Tetanus–Pertussis and Hepatitis B vaccines on vaccination uptake in a health facility in Nigeria. *Vaccine*, 34(24):2722–2728, 2016.

[18] Christopher J Gill, Lawrence Mwananyanda, William MacLeod, Geoffrey Kwenda, Magdalene Mwale, Anna L Williams, Kazungu Siazeele, Zhaoyan Yang, James Mwansa, and Donald M Thea. Incidence of severe and non-severe pertussis among HIV-exposed and-unexposed Zambian infants through 14 weeks of age: results from the southern Africa mother infant pertussis study (SAMIPS), a longitudinal birth cohort study. *Clinical Infectious Diseases*, 63(suppl 4):S154–S164, 2016.

[19] World Health Organization. Pertussis vaccines: WHO position paper—august 2015. *Weekly Epidemiological Record*, 90(35):433–460, 2015.

[20] B Laubereau, M Hermann, HJ Schmitt, J Weil, and R von Kries. Detection of delayed vaccinations: a new approach to visualize vaccine uptake. *Epidemiology & Infection*, 128(2):185–192, 2002.

[21] Zhiyun Chen and Qiushui He. Immune persistence after pertussis vaccination. *Human vaccines & immunotherapeutics*, 13(4):744–756, 2017.

[22] WHO. WHO-UNICEF estimates time series for Zambia (ZMB). http://apps.who.int/immunization_monitoring/globalsummary/estimates?c=ZMB. Accessed: 2019-10-01.

[23] Central Statistical Office/Zambia, Ministry of Health/Zambia, University of Zambia Teaching Hospital Virology Laboratory, University of Zambia Department of Population Studies, Tropical Diseases Research Centre/Zambia, and ICF International. Zambia demographic and health survey 2013-14. http://dhsprogram.com/ pubs/pdf/FR304/FR304.pdf, 2015.

[24] Anna Larson Williams, Rachel Mitrovich, Lawrence Mwananyanda, and Chris Gill. Maternal vaccine knowledge in low-and middle-income countries—and why it matters. *Human vaccines & immunotherapeutics*, 15(2):283– 286, 2019.

[25] Yanping Li, Rong Cheng Li, Qiang Ye, Changgui Li, You Ping Liu, Xiao Ma, Yanan Li, Hong Zhao, Xiaoling Chen, Deepak Assudani, et al. Safety, immunogenicity and persistence of immune response to the combined Diphtheria, Tetanus, acellular Pertussis, poliovirus and Haemophilus influenzae type b conjugate vaccine (dtpaipv/hib) administered in Chinese infants. *Human vaccines & immunotherapeutics*, 13(3):588–598, 2017.

[26] Pamela Rendi-Wagner, Joshua Tobias, Larisa Moerman, Sophy Goren, Ravit Bassal, Manfred Green, and Dani Cohen. The seroepidemiology of Bordetella pertussis in Israel—estimate of incidence of infection. *Vaccine*, 28 (19):3285–3290, 2010.

[27] FMG Magpantay and P Rohani. Dynamics of pertussis transmission in the United States. *American journal of epidemiology*, 181(12):921–931, 2015.

[28] Centers for Disease Control and Prevention. Recommended antimicrobial agents for the treatment and postexposure prophylaxis of pertussis: 2005 CDC guidelines. *MMWR*, 54(RR-14):1–16, 2005.

[29] R.M. Anderson and R.M. May. *Infectious diseases of humans*. Oxford University Press Oxford, 1991.

For peer review only - http://bmjopen.bmj.com/site/about/guidelines.xhtml

[30] Veronica Kolos, Robert Menzies, and Peter McIntyre. Higher pertussis hospitalization rates in indigenous Australian infants, and delayed vaccination. *Vaccine*, 25(4):588–590, 2007.

[31] Helen Campbell, Gayatri Amirthalingam, Nick Andrews, Norman K Fry, Robert C George, Timothy G Harrison, and Elizabeth Miller. Accelerating control of pertussis in England and Wales. *Emerging infectious diseases*, 18 (1):38, 2012.

[32] Md Jasim Uddin, Charles P Larson, Elizabeth Oliveras, AI Khan, MA Quaiyum, and Nirod Chandra Saha. Child immunization coverage in urban slums of Bangladesh: impact of an intervention package. *Health policy and planning*, 25(1):50–60, 2009.

[33] Martin K Mutua, Elizabeth Kimani-Murage, and Remare R Ettarh. Childhood vaccination in informal urban settlements in Nairobi, Kenya: who gets vaccinated? *BMC public health*, 11(1):6, 2011.

Figure Captions

Figure 1. **A:** Study participation by infant age. Shaded regions show scheduled administration of DTP doses 1-3. **B:** Timeline of DTP dose administration (see also Figure S2). C: Number of vaccinations per week, by dose number.

Figure 2. Relative risk of vaccination outcome (columns) by demographic covariate (y-axis), showing 95% CI. Select p-values are shown above estimates. Covariates: @birth indicates the respective vaccine was administered at birth; HIV+ & ART indicates the mother was HIV+ and taking antiretroviral therapy at time of enrollment. See Table 2 for outcome frequencies.

Figure 3. Modified Kaplan-Meier analysis: estimated dose administration probability for DTP doses 1-3, assuming no vaccination in infants lost to follow-up. Vertical grey lines show dose target age; intersections with horizontal dashed lines show the median, upper quantile (slowest 25%) and upper decile (slowest 10%) delays for each dose. Tables show number of infants at select ages.

Figure 4. **A:** Number of early vaccinations (DTP1) by infant age. **B:** Number of vaccinations by dose spacing (time since previous dose), split by dose number (columns). Rows show compressed versus appropriate spacing (rows).

For peer review only

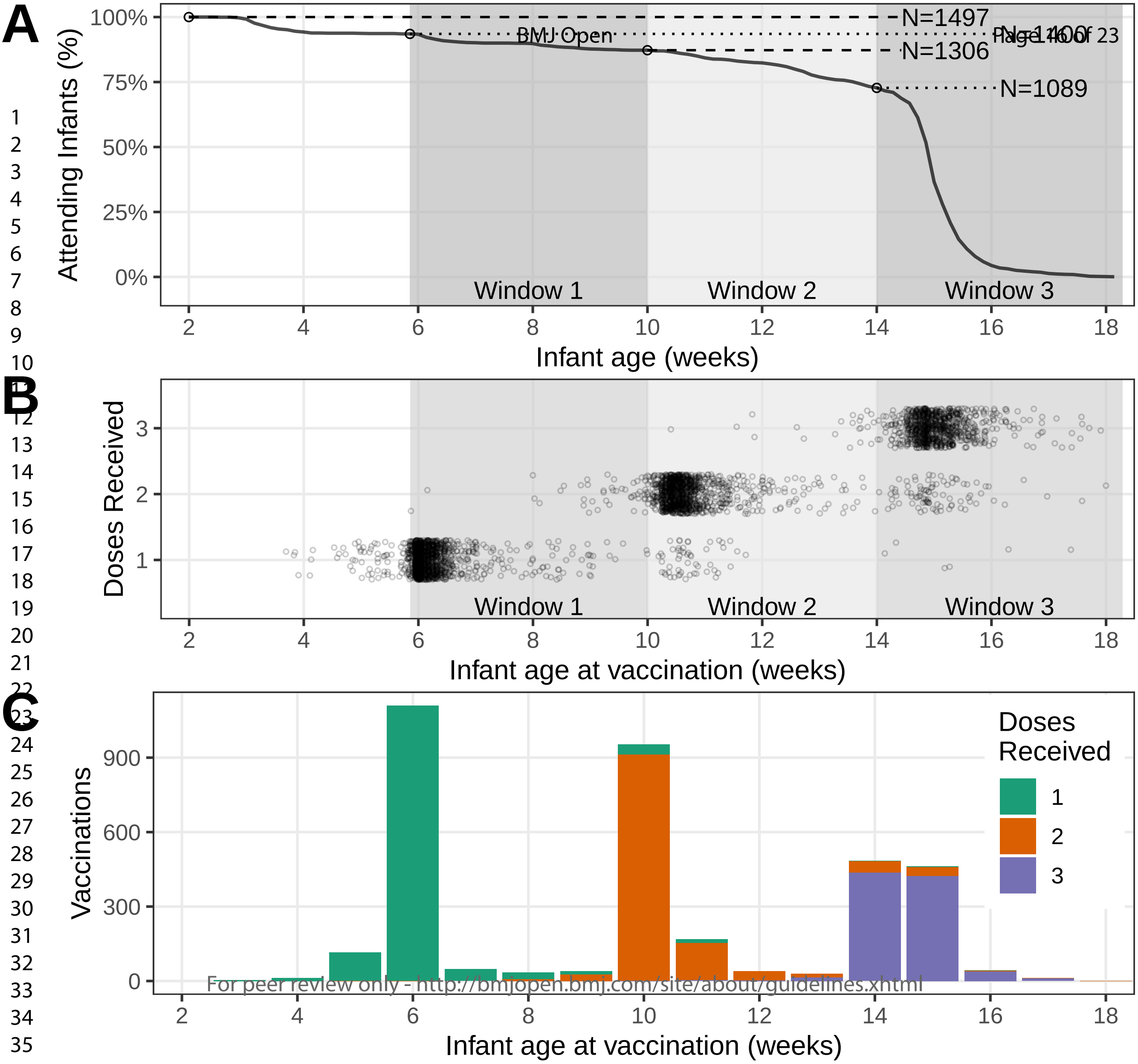
A
Attending Infants (%)
100%
75%
50%
25%
0%
N=1497
N=1400
N=1306
N=1089
Window 1
Window 2
Window 3
2
4
6
8
10
12
14
16
18
Infant age (weeks)
B
Doses Received
3
2
1
Window 1
Window 2
Window 3
Infant age at vaccination (weeks)
C
Vaccinations
900
600
300
0
Doses Received
1
2
3
Infant age at vaccination (weeks)

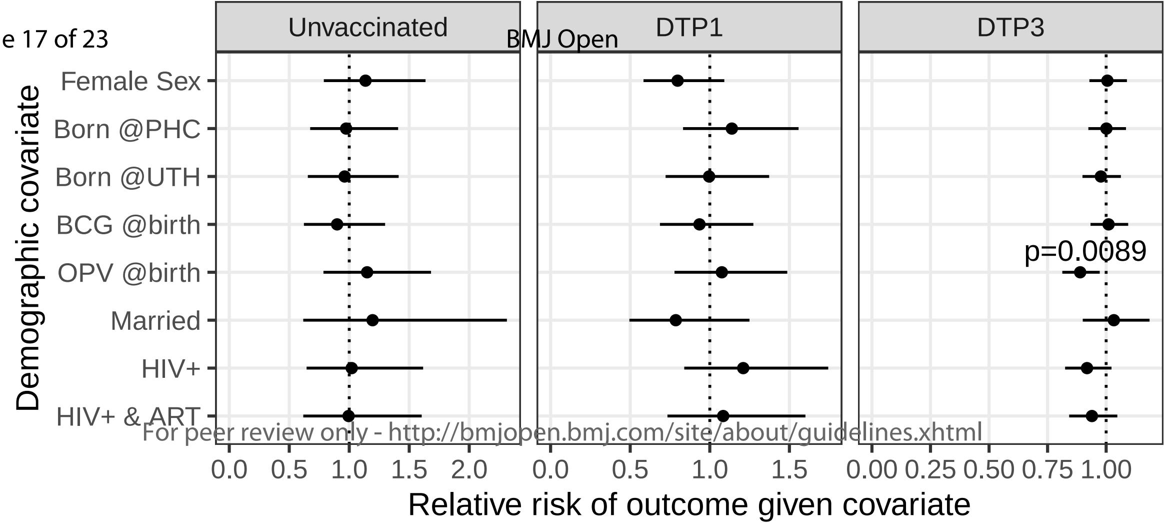
Unvaccinated
DTP1
DTP3
Demographic covariate
Female Sex
Born @PHC
Born @UTH
BCG @birth
OPV @birth
Married
HIV+
HIV+ & ART
p=0.0089
0.0 0.5 1.0 1.5 2.0
0.0 0.5 1.0 1.5
0.00 0.25 0.50 0.75 1.00
Relative risk of outcome given covariate

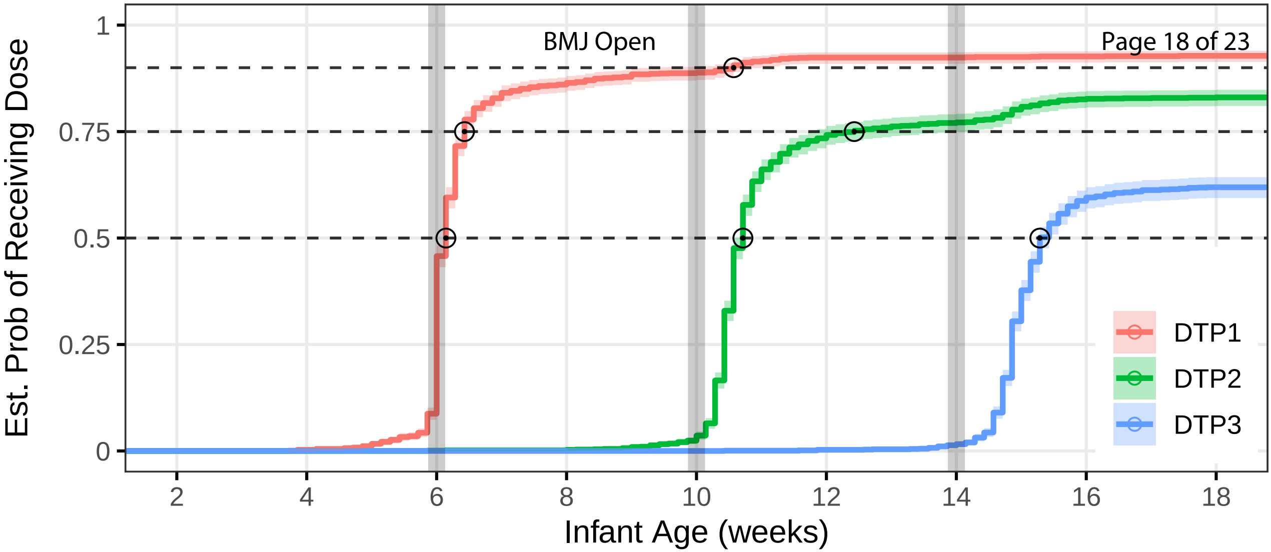

Attending & Eligible:

	2	4	6	8	10	12	14	16	18
DTP1	1497	1412	1276	118	77	12	7	3	1
DTP2	1497	1415	1399	1345	1275	182	111	10	2
DTP3	1497	1415	1400	1347	1310	1230	1086	62	3

Vaccinations (Cumulative):

	2	4	6	8	10	12	14	16	18
DTP1	0	4	685	1294	1330	1383	1383	1387	1389
DTP2	0	0	1	4	54	1111	1155	1238	1243
DTP3	0	0	0	0	0	4	24	891	927

Lost To Future Followup (Cumulative):

	2	4	6	8	10	12	14	16	18
DTP1	0	87	90	91	92	103	107	107	107
DTP2	0	88	115	159	189	218	236	251	253
DTP3	0	88	115	159	191	269	405	558	568

Infant Age (weeks)

For peer review only - http://bmjopen.bmj.com/site/about/guidelines.xhtml

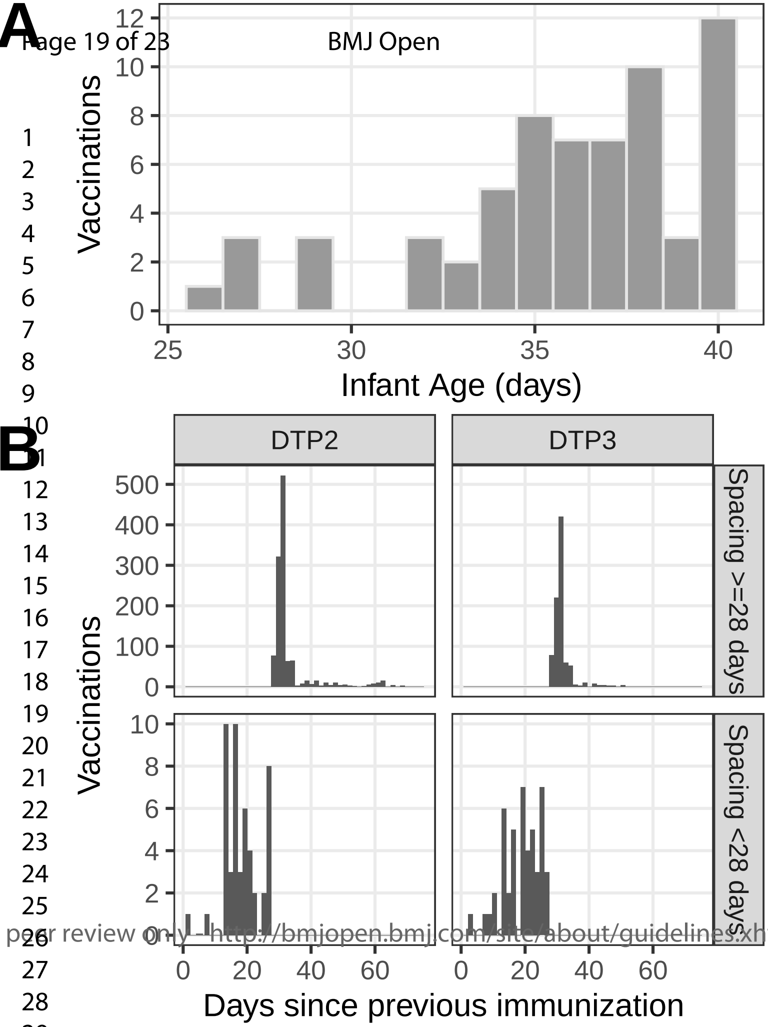

For peer review only - http://bmjopen.bmj.com/site/about/guidelines.xhtml

Implementation and adherence of routine pertussis vaccination (DTP) in a low-resource urban birth cohort

Gunning et al.

November 13, 2020

For peer review only

Supplemental Figures and Tables

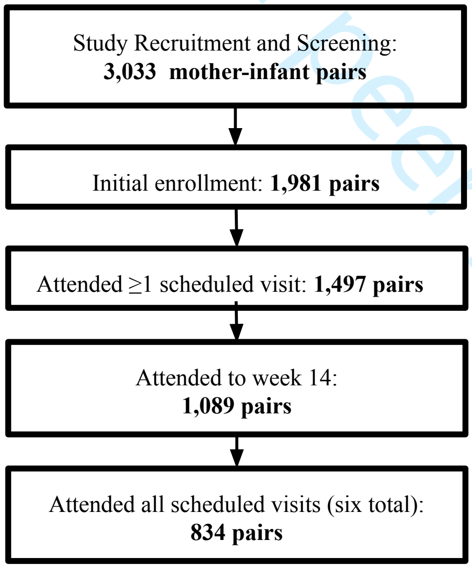

Figure S1: Study profile of birth cohort enrollment and attendance. Beyond eligibility and initial screening, the sole cause of cohort attrition was failure to attend one or more scheduled clinic visits.

For peer review only - http://bmjopen.bmj.com/site/about/guidelines.xhtml

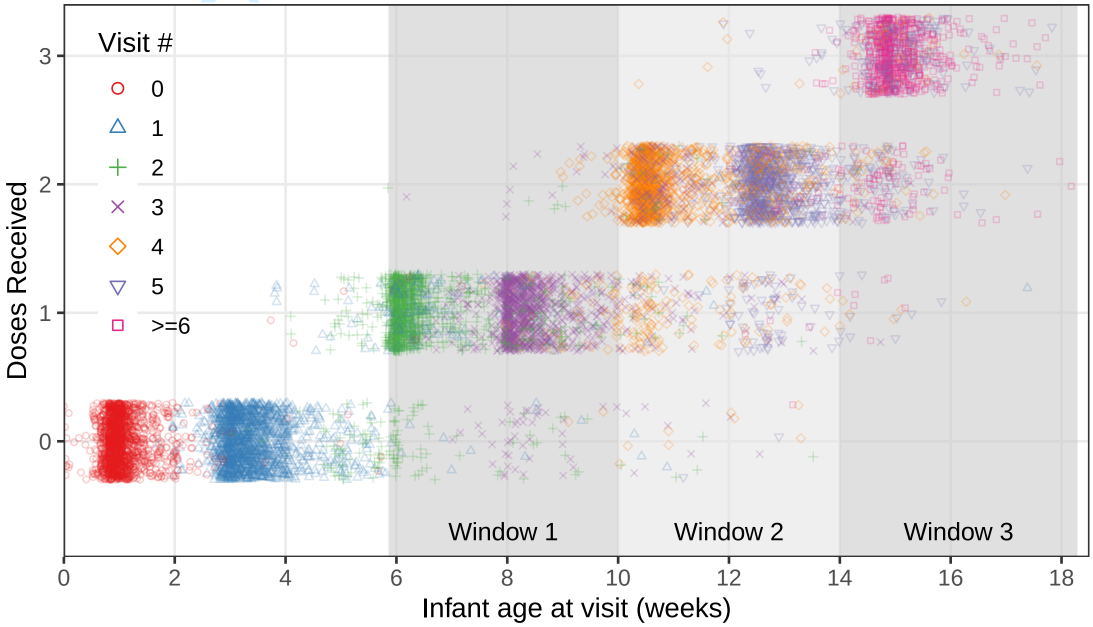

Figure S2: Infants were scheduled for 6 clinic visits over the course of the study. Figure shows the cumulative doses of DTP vaccine received per infant at each visit (color, shape) by infant age.

For peer review only - http://bmjopen.bmj.com/site/about/guidelines.xhtml

STROBE Statement—Checklist of items that should be included in reports of ***cohort studies***

	Item No	Recommendation
**Title and abstract**	1	(*a*) Indicate the study's design with a commonly used term in the title or the abstract<br>**Title, "birth cohort", p1**
		(*b*) Provide in the abstract an informative and balanced summary of what was done and what was found<br>**Abstract, sections Methods and Results, p1**
**Introduction**		
Background/rationale	2	Explain the scientific background and rationale for the investigation being reported<br>**Intro, Paras 1-3, p2**
Objectives	3	State specific objectives, including any prespecified hypotheses<br>**Intro, Paras 3 & 5, p 2-3**
**Methods**		
Study design	4	Present key elements of study design early in the paper<br>**Methods, section Study Design, p 3-4**
Setting	5	Describe the setting, locations, and relevant dates, including periods of recruitment, exposure, follow-up, and data collection<br>**Methods, section Study Design, p3**
Participants	6	(*a*) Give the eligibility criteria, and the sources and methods of selection of participants. Describe methods of follow-up<br>**Methods, section Study Design, p3**
		(*b*) For matched studies, give matching criteria and number of exposed and unexposed<br>**NA**
Variables	7	Clearly define all outcomes, exposures, predictors, potential confounders, and effect modifiers. Give diagnostic criteria, if applicable<br>**Methods, section Assessment, p4**
Data sources/ measurement	8*	For each variable of interest, give sources of data and details of methods of assessment (measurement). Describe comparability of assessment methods if there is more than one group<br>**Methods, section Assessment, p4**
Bias	9	Describe any efforts to address potential sources of bias<br>**Methods, section Data and Statistical Analysis, last para, p5**
Study size	10	Explain how the study size was arrived at<br>**Previously described (citation 18), p3**
Quantitative variables	11	Explain how quantitative variables were handled in the analyses. If applicable, describe which groupings were chosen and why<br>**Methods, section Data and Statistical Analysis, p5**
Statistical methods	12	(*a*) Describe all statistical methods, including those used to control for confounding<br>**Methods, section Data and Statistical Analysis, p5**
		(*b*) Describe any methods used to examine subgroups and interactions<br>**Methods, section Data and Statistical Analysis, p5**
		(*c*) Explain how missing data were addressed<br>**Methods, section Data and Statistical Analysis, p5**
		(*d*) If applicable, explain how loss to follow-up was addressed<br>**Methods, section Data and Statistical Analysis, p5**
		(*e*) Describe any sensitivity analyses<br>**NA**

**Results**		
Participants	13*	(a) Report numbers of individuals at each stage of study—eg numbers potentially eligible, examined for eligibility, confirmed eligible, included in the study, completing follow-up, and analysed<br>**Results, Section Participation, Fig 1A, Table 1, Figure S1, p5-6**
		(b) Give reasons for non-participation at each stage<br>**NA**
		(c) Consider use of a flow diagram<br>**Figure S1**
Descriptive data	14*	(a) Give characteristics of study participants (eg demographic, clinical, social) and information on exposures and potential confounders<br>**Results, Section Demographic Characteristics, Table 1, Figure 2, p6**
		(b) Indicate number of participants with missing data for each variable of interest<br>**Figure 1A, Figure 3**
		(c) Summarise follow-up time (eg, average and total amount)<br>**Results, section Participation, p4-5**
Outcome data	15*	Report numbers of outcome events or summary measures over time<br>**Figure 1 & 3, Tables 2-4, p7-8**
Main results	16	(*a*) Give unadjusted estimates and, if applicable, confounder-adjusted estimates and their precision (eg, 95% confidence interval). Make clear which confounders were adjusted for and why they were included<br>**Figure 1 & 3, Tables 2-4, p7-8**
		(*b*) Report category boundaries when continuous variables were categorized<br>**NA**
		(*c*) If relevant, consider translating estimates of relative risk into absolute risk for a meaningful time period<br>**NA**
Other analyses	17	Report other analyses done—eg analyses of subgroups and interactions, and sensitivity analyses<br>**Figure 4**
**Discussion**		
Key results	18	Summarise key results with reference to study objectives<br>**Discussion, paras 1-3, p 9**
Limitations	19	Discuss limitations of the study, taking into account sources of potential bias or imprecision. Discuss both direction and magnitude of any potential bias<br>**Discussion, paras 4-5, p10**
Interpretation	20	Give a cautious overall interpretation of results considering objectives, limitations, multiplicity of analyses, results from similar studies, and other relevant evidence<br>**Discussion, paras 7-8, p10-11**
Generalisability	21	Discuss the generalisability (external validity) of the study results<br>**Discussion, para 7&9, p10-11**
**Other information**		
Funding	22	Give the source of funding and the role of the funders for the present study and, if applicable, for the original study on which the present article is based<br>**Section Funding, p11**

*Give information separately for exposed and unexposed groups.

Note: An Explanation and Elaboration article discusses each checklist item and gives methodological background and published examples of transparent reporting. The STROBE checklist is best used in conjunction with this article (freely available on the Web sites of PLoS Medicine at http://www.plosmedicine.org/, Annals of Internal Medicine at http://www.annals.org/, and Epidemiology at http://www.epidem.com/). Information on the STROBE Initiative is available at http://www.strobe-statement.org.
